# Assessment of Smartphone-Based Spiral Tracing in Multiple Sclerosis Reveals Intra-Individual Reproducibility as a Major Determinant of the Clinical Utility of the Digital Test

**DOI:** 10.3389/fmedt.2021.714682

**Published:** 2022-02-01

**Authors:** Komi S. Messan, Linh Pham, Thomas Harris, Yujin Kim, Vanessa Morgan, Peter Kosa, Bibiana Bielekova

**Affiliations:** ^1^National Institutes of Health, National Institute of Allergy and Infectious Diseases, Office of Data Science and Emerging Technologies, Rockville, MD, United States; ^2^National Institutes of Health, National Institute of Allergy and Infectious Diseases, Laboratory of Clinical Immunology and Microbiology, Neuroimmunological Diseases Section, Bethesda, MD, United States

**Keywords:** reproducibility, clinical utility, smartphone tests, neurological functions, disability, upper extremity function, dominant and non-dominant hand, multiple sclerosis

## Abstract

Technological advances, lack of medical professionals, high cost of face-to-face encounters, and disasters such as the COVID-19 pandemic fuel the telemedicine revolution. Numerous smartphone apps have been developed to measure neurological functions. However, their psychometric properties are seldom determined. It is unclear which designs underlie the eventual clinical utility of the smartphone tests. We have developed the smartphone Neurological Function Tests Suite (NeuFun-TS) and are systematically evaluating their psychometric properties against the gold standard of complete neurological examination digitalized into the NeurEx^TM^ app. This article examines the fifth and the most complex NeuFun-TS test, the “Spiral tracing.” We generated 40 features in the training cohort (22 healthy donors [HD] and 89 patients with multiple sclerosis [MS]) and compared their intraclass correlation coefficient, fold change between HD and MS, and correlations with relevant clinical and imaging outcomes. We assembled the best features into machine-learning models and examined their performance in the independent validation cohort (45 patients with MS). We show that by involving multiple neurological functions, complex tests such as spiral tracing are susceptible to intra-individual variations, decreasing their reproducibility and clinical utility. Simple tests, reproducibly measuring single function(s) that can be aggregated to increase sensitivity, are preferable in app design.

## Introduction

Expert neurological examination is an art that is slowly but surely disappearing (1). The skilled neurologist can reliably identify deficits in neurological function(s) and localize them to the specific part of the central (CNS) or peripheral nervous system (PNS). An expert examiner can also differentiate deficit that lacks anatomical substrate, by examining identical neurological function in different ways, noting inconsistencies, and motivating a patient to provide adequate effort. Such neurological examination takes between 30 and 60 min to perform and years to master. Because of examiner dependency, the quantitative aspect of neurological examination, especially when performed by different raters, is less precise. Traditional neurological disability scales non-algorithmically aggregate semi-quantitative ratings of different neurological functions, usually selected by an individual [e.g., in Expanded Disability Status Scale, EDSS; (2)] or teams of experts [e.g., in Scripps Neurological Rating Scale, SNRS; (3)], into a single number. This is suboptimal for two reasons: (1) the features of the neurological examination aggregated to the disability scale are not data-driven and therefore may not be optimal and (2) the lack of a defined algorithm causes errors during the translation of the examination into a scale. These drawbacks are eliminated by data-driven scales [such as Combinatorial Weight-Adjusted Disability Scale, CombiWISE; (4)] and digital tools that allow convenient documentation of neurological examination in its entirety with automated, algorithmically codified computation of relevant disability scales [such as NeurEx^TM^ app; (5)].

However, these solutions are useless when the lack of expert medical professionals, limited time for patient encounters, or inability to examine patients in person due to pandemics deprives patients of the benefit of this historically validated tool. Therefore, there is a strong movement to supplement neurological examination or, in some instances, to replace it, by patient-autonomous tests of neurological functions (both cognitive and physical) acquired via smartphones, tablets, or web interphase (6–15).

While some of these apps are already marketed to patients, they often lack careful assessment of their psychometric properties against the gold standard of neurological examination and imaging or electrophysiological measures of CNS (or PNS) injury. Even a simple assessment of test-retest reproducibility may be missing. For instance, while the work of Creagh et al. (9) demonstrated the potential of the smartphone-based test to predict 9-Hole Peg Test (9HPT) in the training cohort of subjects with multiple sclerosis (MS), no evaluation of test-retest reproducibility (or accuracy of 9HPT prediction in the independent validation cohort) was provided.

Many of these apps use tests adopted from standard neurological examination and modified to self-administered digital tests. This is true for the Spiral tracing test examined in this paper. Spiral tracing has been used in movement disorders to identify tremors and quantify their severity. Its digitalization offers automated identification and quantification of the tremor frequency and amplitude by Fourier transformation (16). Furthermore, digitalization of the shape(s) tracing allows other quantitative measurements of speed and precision of the tracing (by finger or stylus) which may reflect neurological (dys)functions.

We have reviewed previous studies of digitalized spiral/object tracing (9–11, 17) to derive a comprehensive set of digital features (40 total) and determined their psychometric properties (i.e., reproducibility, ability to differentiate patients with MS from healthy donors (HD) and correlation with relevant features of neurological examination, disability scales and CNS tissue destruction visible on brain MRI) in the training and independent validation cohorts of patients with MS. We hypothesized that by virtue of aggregating multiple neurological functions (i.e., vision, fine finger motoric, proprioceptive and cerebellar functions) in the test performance, spiral tracing will outperform simpler smartphone tests that we have evaluated previously in the Neurological Function Tests Suite (NeuFun-TS), such as finger or foot tapping, balloon popping, and level test, which demonstrated comparable or even stronger sensitivity and specificity to traditional non-clinician-acquired disability measures, such as 9HPT (7, 18, 19). Specifically, finger tapping, a simple motoric test consisting of tapping a finger on the surface of a smartphone for 10 s as rapidly as possible achieved Pearson correlation coefficients of up to 0.75 with NeurEx-derived cerebellar functions, 0.73 with motoric functions, and 0.69 for strength subscore of the motoric functions. Analogous correlations were observed for the balloon popping test, where a subject was required to tap a balloon that randomly appeared at different locations of the smartphone screen (i.e., balloon popping test). The level test, where a subject is tilting smartphone screen to guide a “ball” that appears at random locations at the periphery of the smartphone to the designated center of the screen and holds the ball in the center during the test achieves Spearman correlations of up to 0.4 with proprioception, 0.42 with motor functions, 0.49 with muscle atrophy subscore of motor functions, and 0.63 with cognitive functions.

Because we were unsure of the optimal size/thickness of the spiral in the spiral-tracing digital adaptation, we tested three different levels of increasing difficulty. However, contrary to our expectation, we observed comparatively weak correlations (Spearman Rho up to 0.33) of spiral-tracing-derived outcomes with simultaneously measured features from neurological examination documented in the NeurEx^TM^ app. Furthermore, intra-individual test reproducibility and correlation of the best spiral-tracing outcome (the sum of Hausdorff distances) with the clinician-derived disability outcomes *decreased* with the increasing test difficulty, leading us to conclude that the poor clinical utility of the spiral-tracing outcomes is due to their poor intra-individual reproducibility.

Because other authors (9) aggregated multiple features from the spiral-tracing test to achieve a stronger correlation with traditional disability outcomes such as 9HPT using supervised machine-learning (ML) algorithms, we performed the same analyses here. We reproduced the ability to derive models with strong cross-validation performance in the training cohort (i.e., *R*^2^ up to 0.73 for correlation with 9HPT). In contrast to the performance of the best spiral tracing outcome (i.e., the sum of the Hausdorff distances) the cross-validation performance of the ML models in the training cohort *increased* with the increasing test difficulty. However, this apparent increase in the performance of ML models was entirely due to overfitting; when applied to the true independent validation cohort, all ML models performed poorly so that none outperformed the Hausdorff distances.

## Materials and Methods

### Participants

The data were collected from participants enrolled in the Natural History protocol: Comprehensive Multimodal Analysis of Neuroimmunological Diseases in the Central Nervous System (ClinicalTrials.gov identifier NCT00794352). The study was approved by the National Institute of Allergy and Infectious Diseases (NIAID) scientific review and by the National Institutes of Health (NIH) Institutional Review Board. All methods were performed in accordance with the relevant guidelines and regulations. All study participants gave informed consent. HD was recruited in two ways: (1) full participants in the Natural History protocol that underwent comprehensive neurological/imaging evaluation and (2) participants in a substudy of the Natural History protocol to obtain normative data for smartphone apps (without neurological/imaging evaluation). Two different groups are comprised within the MS datasets: a cohort that is tested in a clinic approximately every 6 months (non-granular testing sub-cohort) and those that had the smartphone at home and did the test more than 5 times during a period of 2 years (granular testing sub-cohort). Prior to all analyses, the MS datasets were separated into a 2/3 training and 1/3 test set weighted by one of the clinical features (average 9HPT; see Table 2). A summary of the demographic information is provided in Table 1.

**Table 1:** Demographics and characteristics for healthy donors (HD) and patients with multiple sclerosis (MS) over the 2 years of the study period.

**Demographic**	**HD (No. = 22)**	**MS (No. = 134)**		***P*** **-value for Statistical Significance**
		**Training set** **(No. = 89)**	**Test set (No. = 45)**	
Mean age (± SD)	36.7 ± 12.3	56.7 ± 9.48	53.5 ± 8.85	<0.001^K−W^
Median age	39	58	53	NA
Range of age	20–62	19–73	27–78	NA
Gender (Male/Female)	8/14	34/55	19/26	<0.001^χ^
Handedness (Left/Right)	2/20	11/78	2/43	<0.001^χ^
Disease duration (in years)	NA	15.9 ± 10.31	17.9 ± 12.19	NA
No. of treated at the first visit	NA	67	35	NA
No. of untreated at the first visit	NA	22	10	NA

*SD indicates standard deviation from the mean. K-W indicates Kruskal–Wallis non-parametric test for the mean comparison of age across HD, MS-Training set, and MS-Test set. χ denotes the Chi-Square test of independence of the differences between categorical groups (i.e., gender or handedness in HD, MS-Training set, and MS-Test set). NA, No., and MS denote not applicable, number, and multiple sclerosis patients, respectively*.

### Test Design and Data Collection

The Spiral test was written in Java and Kotlin using the Android Studio integrated development environment. The test is distributed as an Android Package (APK) over email, or directly installed to the device over USB, and updates are sent out over the air. The testing devices are Google Pixel XL and Google Pixel 2 XL, running Android (Android Version 11), with the intent of keeping them up to date. Results are uploaded to Firebase Firestore, a commercial cloud database, with alphanumeric identifiers to avoid Personally Identifiable Information. Spirals are generated using physical dimensions and rendered using the individual device's screen characteristics and configuration, to ensure that spirals with the same parameters look the same across all devices.

**Figure 1:**
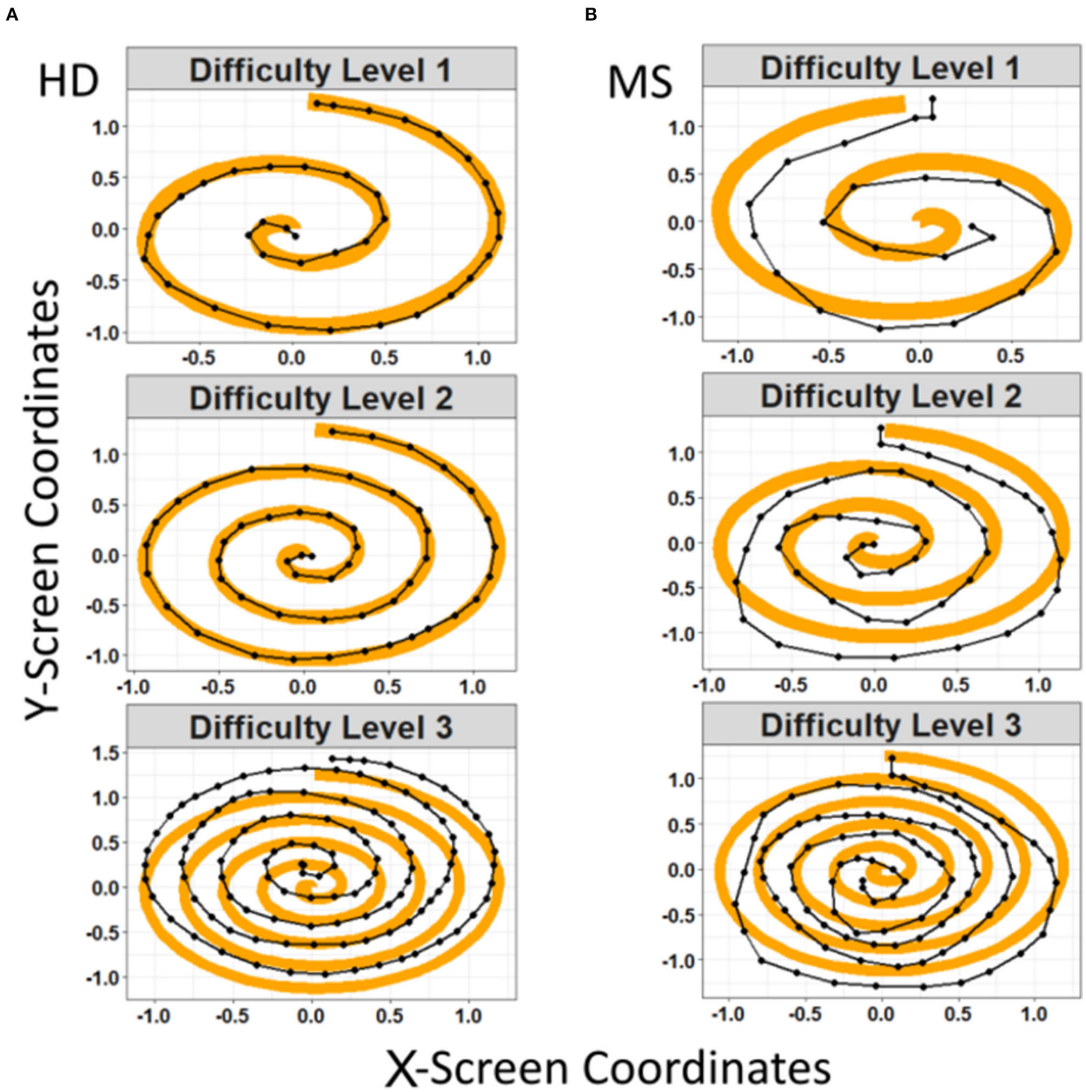
Representation of the spiral tests performed on the smartphone by healthy donors **(A)** and patients with MS **(B)**. The orange and black spirals represent respectively the reference shape and the patient's drawn shape (MS indicates multiple sclerosis patients, HD indicates healthy donors).

Spiral tracing test consisted of tracing with a finger of each hand an orange spiral shown on the screen of the smartphone at three difficulty levels: Level 1 (simplest) consisted of the thickest spiral of shortest length, while level 3 (most difficult) consisted of the thinnest spiral of longest length (Figure 1). Each participant was instructed to trace the spiral as accurately and fast as possible. A total of four test trials were conducted by the subjects at each of the test dates for each difficulty level. Two of the drawings are done clockwise from and to the center of the spiral by the dominant hand and similarly, two drawings are done by the non-dominant hand counterclockwise (i.e., from and to the center of the spiral). Thus, a total of four tests were conducted by each of the subjects on their testing dates. As previously stated, the non-granular testing sub-cohort was tested approximately every 6 months while the granular testing sub-cohort was tested more frequently during the period of 2 years. Hence, the number of tests conducted by each subject throughout the 2 years of study period varies per subject. During the experiment, raw sensor data was collected from the smartphone touchscreen as *x*- and *y*-screen coordinates with a corresponding timestamp in milliseconds and an estimated pressure of the tap based on the surface area of the finger on the touchscreen.

### Clinical Assessments of Motor Symptoms

The complete neurological examination, lasting 30–60 min and performed by an MS-trained clinician was transcribed into the NeurEx^TM^ app (5). NeurEx^TM^ computes traditional disability scales such as EDSS (2), SNRS (3), and others. We also extracted relevant subsystem scores of those neurological functions that, based on domain expertise, contribute to the spiral tracing (i.e., pyramidal and motor functions of hands, cerebellar functions, proprioception functions). Finally, we extracted semi-quantitative MRI data of CNS tissue destruction, focusing on the brainstem, cerebellum, and medulla/upper cervical spinal cord. These are features previously validated as important in determining physical disability (20, 21). The details of MRI sequences and computation of selected MRI features have been previously published (20, 21).

Thus, together we tested 22 disability features in the MS training set (Table 2). Though spiral data were obtained from 22 HD, we note that clinical features were generated for only 9 HD (see Section Participants for details). We highlight that the clinical features extracted from the NeurEx^TM^ app are later used to validate features obtained from the spiral tracing test.

**Table 2:** List of clinical disability scales used in the study.

**Label**	**Feature Description**	**Mean**	**Standard Deviation**	**Statistically Significant**
C1	9HPT average	52.930	106.382	***
C2	Non-dominant hand 9HPT	58.528	142.561	***
C3	Dominant hand 9HPT	47.332	112.122	***
C4	Expanded disability status scale (EDSS; 0-10; ordinal)	4.961	1.692	***
C5	CombiWISE (0-100; continuous)	41.147	15.989	***
C6	NeurEx (0-1349; continuous)	137.041	86.927	***
C7	EDSS visual function score	1.629	1.185	***
C8	NeurEx vision score	3.810	3.485	0.001**
C9	EDSS pyramidal functions score	2.707	1.206	***
C10	NeurEx pyramidal/motor functions non-dominant hand	4.858	4.811	***
C11	NeurEx pyramidal/motor functions dominant hand	4.698	4.875	***
C12	EDSS cerebellar functions score	2.478	1.509	***
C13	NeurEx cerebellar functions non-dominant hand	2.996	2.835	***
C14	NeurEx cerebellar functions dominant hand	2.358	2.461	***
C15	NeurEx vibration and proprioception non-dominant hand	35.315	8.755	***
C16	NeurEx vibration and proprioception dominant hand	34.642	8.656	***
MRI1	Brainstem atrophy	0.703	0.791	0.004**
MRI2	Medulla/Upper C-spine atrophy	0.772	0.829	0.002**
MRI3	Cerebellum atrophy	0.616	0.747	0.007**
MRI4	Lesion load brainstem	1.737	0.919	***
MRI5	Lesion load medulla	1.754	1.009	***
MRI6	Lesion load cerebellum	1.246	0.983	***

** and *** indicate clinical disability scales (i.e., clinical features) that have statistically significant differences between HD and MS at the Benjamini–Hochberg (BH) adjusted p-value of 0.01 and 0.001, respectively, using the unpaired Two-Samples Wilcoxon test. P-values of the Wilcoxon test are provided when statistical significance is found at 0.01 and marked *** when p-value < 0.001. 18 out of the 22 clinical features were statistically significant between HD and MS at a p-value < 0.001. HD and MS indicate healthy donors and multiple sclerosis patients, respectively.

### Data Processing and Analysis

#### Feature Extractions

The raw sensor data was processed with signal and time series analysis methodologies to compute temporal, spatial, and spatiotemporal features. When appropriate, features were calculated following the work of Creagh et al. (9) in addition to some new features computed in this work and this generated a total of 40 spiral-derived features (i.e., digital features). To measure temporal irregularities in the upper extremity function in neurological patients, previous research used speed and velocity as signals in the objective quantification of motor symptoms (9, 22–24). Thus, we initially computed the velocity (v), radial velocity, and angular velocity (av) of the drawing spirals as follows:


(1) $$\begin{array}{r} v=\frac{\sqrt{{\left(x_{i+1}-x_{i}\right)}^{2}+{\left(y_{i+1}-y_{i}\right)}^{2}}}{t_{i+1}-t_{i}} \end{array}$$

Where *x*_*i*_, *y*_*i*_, *i* = 1…*N* are the horizontal and vertical coordinates of pixels on the screen respectively with *N* representing the total number of touch data points.*t*_*i*_,*i* = 1…*N* is the timestamp converted to second. The radial velocity is computed as follows:


(2) $$\begin{array}{r} rv=\frac{r_{i+1}-r_{i}}{t_{i+1}-t_{i}} \end{array}$$

Where $r=\sqrt{\left(x^{2}+y^{2}\right).}$ If we denote θ the four-quadrant inverse tangent $\left(i.e.,\;\theta =\tan^{-1}\left(\frac{y}{x}\right)\right),\;$then the angular velocity takes the following form:


(3) $$\begin{array}{r} av=\frac{\theta_{i+1}-\theta_{i}}{t_{i+1}-t_{i}} \end{array}$$

The sum, coefficient of variation, skewness, and kurtosis were computed for the velocity, radial velocity, and angular velocity, respectively.

To calculate the degree of resemblance between the reference spiral and cohort's drawing, we introduced features related to the Hausdorff distance, which quantify the extent to which each point in the reference spiral lies near the points in the cohort's drawing following procedures illustrated (9, 25–27). Similar to Figure 3 in Jeong and Srinivasan (27), a detailed example procedure to calculate the Hausdorff distance of the reference and cohort's drawing is presented in Supplementary Figure S1. We point out that the Hausdorff distance was calculated using the “metric.hausdorff” function in the fda.usc R software package (28). Prior to calculating the Hausdorff distance, the *x* and *y* screen coordinate points of the reference spiral were interpolated to the length of the cohort's drawing's coordinates using cubic spline interpolation (29–31). Several Hausdorff distance-related features were then calculated (e.g., maximum of Hausdorff distance, interquartile range of Hausdorff distances, etc.). All Hausdorff distance related features are provided in Table 3.

**Table 3:** List of digital features relating to upper extremity function calculated from the spiral drawing test.

**Label**	**Feature Description**	**Difficulty Levels (Dominant)**			**Difficulty Levels (Non-dominant)**		
		**1**	**2**	**3**	**1**	**2**	**3**
F1	Sum of velocity	***	***	0.012*	0.009**	0.622	0.285
F2	Coefficient of variation of velocity	***	0.034*	0.157	0.309	0.954	0.549
F3	Skewness of velocity	0.092	0.944	0.459	0.282	0.207	0.012*
**F4**	**Kurtosis of velocity**	***	***	***	***	***	***
F5	Sum of radial velocity	0.254	0.132	0.265	0.341	0.622	0.380
**F6**	**Coefficient of variation of radial velocity**	***	***	***	***	***	***
**F7**	**Skewness of radial velocity**	***	***	***	***	***	***
**F8**	**Kurtosis of radial velocity**	***	***	***	***	***	***
F9	Sum of angular velocity	***	***	***	0.004**	0.001**	0.258
**F10**	**Coefficient of variation of angular velocity**	***	***	***	***	***	***
**F11**	**Skewness of angular velocity**	***	***	***	***	***	***
**F12**	**Kurtosis of angular velocity**	***	***	***	***	***	***
**F13**	**Sum of estimated pressure**	***	***	***	***	***	***
F14	Maximum power spectral density (PSD) of velocity	0.134	0.954	0.934	0.059	0.221	0.580
**F15**	**Dominant frequency of velocity**	***	***	***	***	***	***
F16	Maximum PSD of radial velocity	***	***	0.003**	***	0.020*	0.922
F17	Dominant frequency of radial velocity	0.134	0.388	0.006**	0.259	0.024*	0.158
F18	Maximum PSD of angular velocity	0.805	***	***	0.002**	***	***
F19	Dominant frequency of angular velocity	0.104	0.426	0.361	0.020*	0.177	0.273
**F20**	**Approximate entropy of velocity**	***	***	***	***	***	***
F21	Approximate entropy of radial velocity	***	***	***	***	0.007**	0.166
F22	Approximate entropy of angular velocity	0.099	***	***	***	***	***
F23	Maximum Hausdorff Distance (HDis)	0.003**	***	***	***	0.004**	***
**F24**	**Sum of HDis**	***	***	***	***	***	***
**F25**	**Sum of HDis divided by the time taken to complete drawing**	***	***	***	***	***	***
**F26**	**Interquartile range of sum of HDis**	***	***	***	***	***	***
F27	Sum of HDis normalized by touchpoints at the beginning	0.044*	0.037*	0.336	***	0.003**	0.012*
F28	Sum of HDis normalized by touchpoints at the end	***	***	***	0.019*	***	***
**F29**	**Sum of HDis in the middle 15–85%**	***	***	***	***	***	***
**F30**	**Sum of HDis in the middle 15–85% normalized by the time taken to complete drawing**	***	***	***	***	***	***
F31	Error calculated using area under the curve	***	0.239	***	0.003**	0.261	0.003**
F32	Mean square error	0.001**	0.944	***	***	0.115	0.006**
F33	Root mean square error	0.001**	0.944	***	***	0.115	0.006**
**F34**	**Center of shoot**	***	***	***	***	***	***
**F35**	**Time taken to complete drawing**	***	***	***	***	***	***
F36	Total asymmetry of patient drawing	***	0.016*	0.605	***	***	0.002**
F37	True asymmetry in comparison with a reference shape	***	0.406	0.698	***	0.177	0.052
F38	2D image correlation between two images	***	0.239	0.946	***	0.650	0.358
F39	Image entropy of shape drawn	***	***	***	***	***	***
F40	Image entropy of shape drawn with respect to reference shape	0.149	0.176	0.035*	0.058	0.127	0.625

*, **, and *** indicate features that are statistically significant differences between HD and MS at the Benjamini–Hochberg (BH) adjusted p-value of 0.05, 0.01, and 0.001, respectively, using the unpaired Two-Samples Wilcoxon test. The number in the tables are the p-values of the Wilcoxon test when statistical significance is found at 0.05 or 0.01 and marked *** when p-value < 0.001. The label and feature description of features that are statistically significant at p-value < 0.001 between HD and MS at both the dominant and non-dominant hands and all difficulty levels are bolded. HD and MS indicate healthy donors and multiple sclerosis patients, respectively.

Two approaches were utilized to compute the error-related features between the reference spiral and the cohort's drawings. The first error was computed using the trapezoid to integrate the two spiral regions. This error was calculated by finding the intersection of the two-spiral region (i.e., the difference between the two areas in magnitude). For instance, we note the following trapezoidal formula from Aghanavesi et al. (22):


(4) $$\begin{array}{c} \overset{x_{n}}{\underset{x_{n+1}}{\int^{\text{​}}}}f(x)dx=\frac{b-a}{2N}\overset{N}{\underset{n=1}{\sum^{\text{​}}}}[f(x_{n})-f(x_{n+1})] \end{array}$$

Where *N* is the total number of x or y screen coordinate points and $\frac{b-a}{N}$ is the spacing between points. Let us suppose that the reference spiral and the cohort's spiral are denoted by *f*_*ref*_(*x, y*) and *f*_*coh*_(*x, y*), respectively. Then the error based on the trapezoidal rule becomes


(5) $$\begin{array}{r} AUC\left(x,y\right)=|\overset{x_{n+1}}{\underset{x_{n}}{\int }}f_{ref}\left(x\right)dx-\overset{x_{n+1}}{\underset{x_{n}}{\int }}f_{coh}\left(x\right)dx| \end{array}$$

Where |.| is the absolute value of the difference between the two Area Under the Curve (AUC). We now proceed with the second error calculated using the following two-dimensional (2D) Mean Square Error [MSE; (32)]:


(6) $$\begin{array}{c} nMSE=\frac{1}{M\times N}\overset{M}{\underset{n=1}{\sum^{\text{​}}}}\overset{N}{\underset{m=1}{\sum^{\text{​}}}}{\left[f_{ref}(x,y)-f_{coh}(x,y)\right]}^{2} \end{array}$$

Where *M* and *N*=*2* are the numbers of data points and coordinates points, respectively. Again, a spline interpolation was used on the cohort's data point to the M length of the reference data point prior to error calculation. Furthermore, to obtain the similarity between the reference spiral and cohort's drawing, the following 2D correlation coefficient from Aljanabi, Hussain, and Lu (33) was utilized:


(7) $$\begin{array}{l} corr(IM_{ref},IM_{coh})\\ =\frac{\sum_{m=1}^{M}\sum_{n=1}^{N}\left(A_{MN}-\bar{A})(B_{MN}-\bar{B}\right)}{\sqrt{\left(\sum_{m=1}^{M}\sum_{n=1}^{N}{\left(A_{MN}-\bar{A}\right)}^{2})(\sum_{m=1}^{M}\sum_{n=1}^{N}{\left(B_{MN}-\bar{B}\right)}^{2}\right)}} \end{array}$$

Where *A*_*MN*_ and *B*_*MN*_ are the reference and cohort's spiral coordinate points with dimension *M x 2*, respectively. $\bar{A}=\frac{\sum_{i}x_{i}+\sum_{i}y_{i}}{2M}$ and $\bar{B}=\frac{\sum_{i}x_{i}+\sum_{i}y_{i}}{2M}$ are the reference and cohort's spiral mean. Note that the the reference and cohort's spiral mean (i.e., Ā and $\bar{B}$) are not necessarily equal as the *x* and *y* coordinate points differ. A full list of features calculated is provided in Table 3. When applicable, references of the calculated spiral-derived features are provided in Supplementary Table S1 in the supplemental material.

#### Statistical Analysis

Statistical analyses were used to evaluate the validity and strength of features (i.e., clinical disability scales and spiral-derived features) on assessing the upper extremity function in patients with MS. The analyses were conducted using the R software [R Version 4.0.4; (34)]. Recall that the MS datasets were separated into a 2/3 training and 1/3 test set weighted by the average 9HPT disability scale. A cutoff Benjamini–Hochberg [BH; (35)] adjusted *p*-value < 0.001 was used to establish statistically significant differences for comparing the HD and MS cohorts. All features that were not statistically significant using the unpaired Two-Samples Wilcoxon test (36) in the training set were removed from subsequent analysis. Moreover, average fold change (FC) between the HD and MS was computed at all difficulty levels and at both dominant and non-dominant hand. An FC > 2 was used as cutoff of significant difference between HD and MS.

Test-retest reliability of the spiral-derived features was measured using the intraclass correlation coefficient [ICC; (15)] of features obtained from the granular testing HD and MS sub-cohorts. The ICC was calculated using the ICC function from the irr R package (37). As stated by (38, 39), there are several versions of the ICC that can give different results when used on the same dataset. However, the authors pointed that the two-way mixed-effects model and the absolute agreement are more appropriate for test-retest reliability studies. Thus, an ICC with two-way mixed-effects model and the absolute agreement was used in this study. Following the recommendation of Koo and Li (38) that stated that an ICC between 0.5 and 0.75 are considered moderate, a Spearman correlation matrix between clinical disability scales (see Table 2) and spiral-derived features (see Table 3) with ICC > 0.5 and FC > 2 were constructed. A BH-adjusted *p*-value > 0.05 was used to access the statistical significance of the correlation test.

To determine the existing relationship between the significant spiral-derived features (i.e., BH-adjusted *p*-value < 0.001, FC > 2, and ICC > 0.5 between HD and MS) and the statistically significant clinical disability scales, four different regression models (Elastic Net or ElasticNet, Support Vector Regression with Radial Basis Function Kernel or SVR Radial, Random Forest or RF, and Stochastic Gradient Boosting or GBM) were used where the clinical disability scale were the dependent variables while spiral-derived features were the independent variables. For all regression models, the “caret” library (40) in the R software was used along with other libraries such as “glmnet” (41) for ElasticNet model, “randomForest” (42) for RF model, and “xgboost” (43) for GBM model. Prior to the regression modeling, outliers in the spiral-derived features were identified as feature values that lie outside of ± 2(q_0.9_ –q_0.1_) where q_p_ is the p-quantile (44). To reduce variability in the features, all variables were bi-symmetric log-transformed using the transformation formula *y* = *sgn*(*x*)log_10_(1+|*x*/*C*|) where *y* is the transformed function of the *x* variable, C has a default value of 1/ln (10), and *sgn(x)* is the mathematical Signum function [as presented in (45)].

Moreover, a linear regression model was used to assess the relationship between selected clinical disability scales and the sum of the Hausdorff distances (Feature F24 in Table 3). During the analysis, adherence to the normality assumptions of the residuals was tested using histograms and quantile plots. All models were evaluated using the Root Mean Square Error (RMSE; measured in seconds) and the coefficient of determination (R^2^) of the prediction. Apart from the linear regression model, all model parameters (i.e., the penalty strength parameter λ and the penalties from both L1 and L2 regularization parameter α in ElasticNet; the cost value C and γ of the SVR Radial; the number of variables randomly sampled at each split in the RF model; the number of trees, the interaction depth, the minimum number of samples in tree terminal nodes, and the learning rate in GBM model) were tuned via grid search. Five-fold cross-validation (CV) with 10 repetitions was used to assess the model suitability in the training cohort. The out-of-sample test performance was evaluated using the final model from the 5-fold CV based on the RMSE to predict clinical disability scales given the test datasets (i.e., the independent validation cohort).

## Results

### Feature Evaluation

To determine clinical disability scales and spiral-derived features that are relevant for further analysis, statistical significance between HD and MS was calculated using the unpaired Two-Samples Wilcoxon test. With the exception of four features (NeurEx^TM^ vision score, brainstem atrophy, medulla/upper c-spine atrophy, and cerebellum atrophy), all clinical disability scales and MRI features were found to be statistically significant at *p* < 0.001 after adjusting the *p*-value using the BH approach (Table 2). There were differences in spiral-derived features that were statistically significant between the dominant and non-dominant hands. In the dominant hand category, for instance, spiral-derived features 21 and 28 were statistically significant (BH adjusted *p*-value < 0.001) at the difficulty level 1, 2, and 3. However, in the non-dominant hand category, these features (i.e., 21 and 28) were not statistically significant at any difficulty levels. We also found spiral-derived feature 22 to be statistically significant at all difficulty levels in the non-dominant hands (BH adjusted *p*-value < 0.001) but not in the dominant hands. While spiral-derived features that were statistically significant between HD and MS vary between dominant, non-dominant hands and difficulty levels, 19 of these features were consistently statistically significant at all levels and both hands (see bold feature description in Table 3).

**Figure 2:**
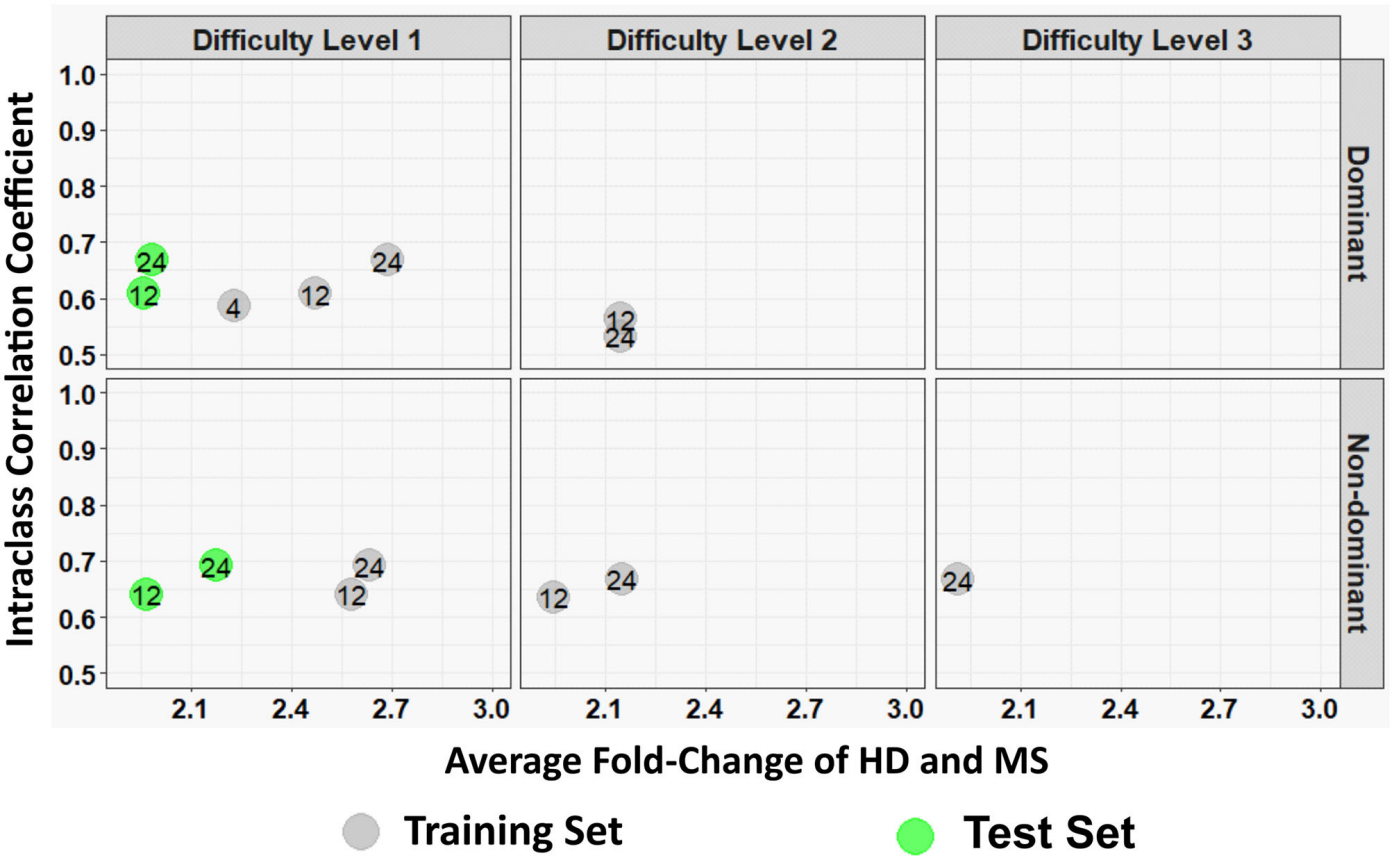
Average fold change (FC) of HD and MS of the spiral-derived features with respect to their Intraclass Correlation Coefficient (ICC) where ICC was calculated from the granular data of HD. The numbers indicate the feature's labels as illustrated by the label in Table 2. Gray colors are features from the training set while green colors are from the test set. Here we only include FC > 2 and ICC > 0.5. A diagram of all features that are statistically significant between HD and MS in the training and test set for HD ICC is provided as Supplementary Figures S5, S6, respectively in the Supplementary Material (HD and MS indicate healthy donors and multiple sclerosis patients, respectively).

**Figure 3:**
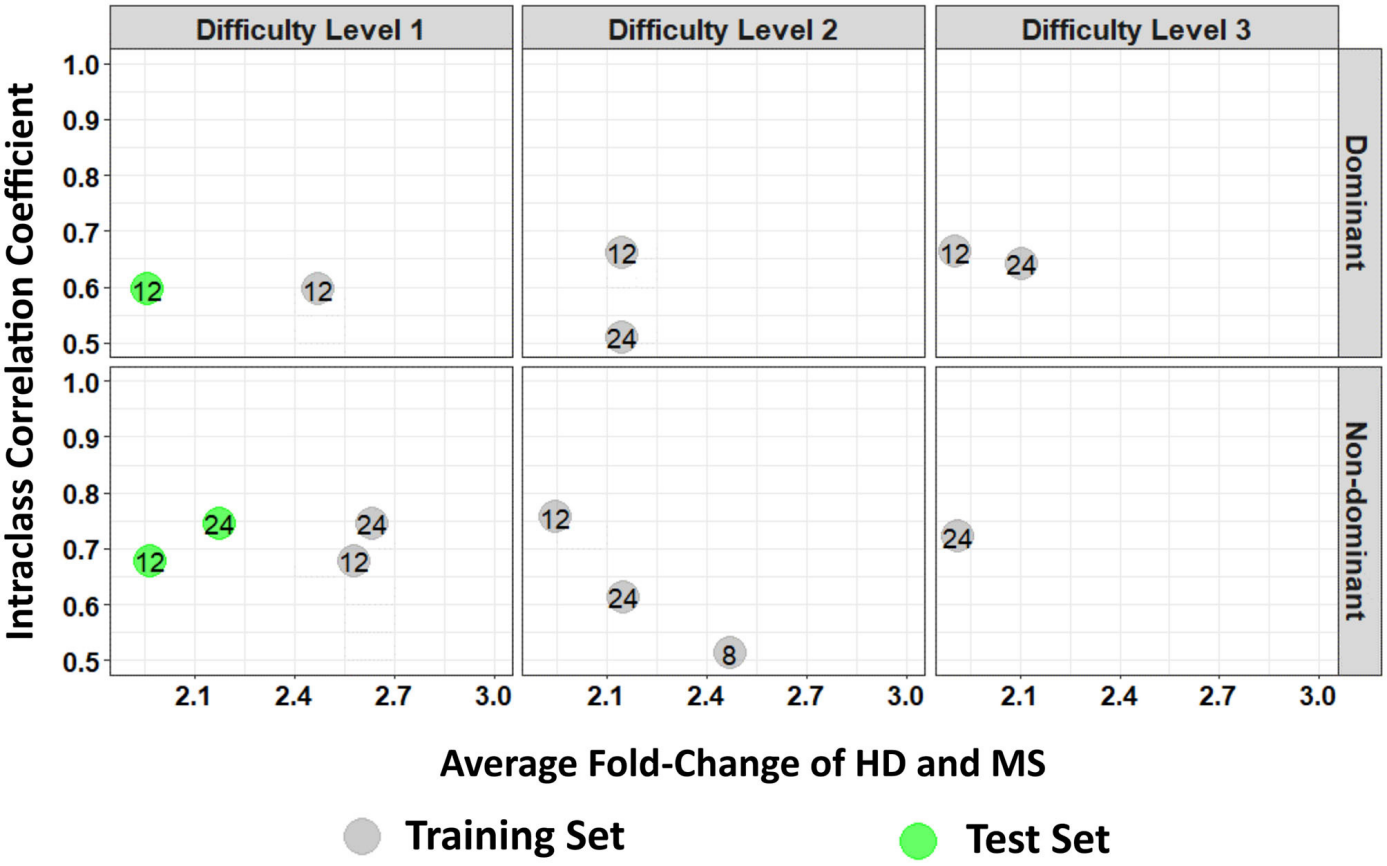
Average fold change (FC) of HD and MS of the spiral-derived features with respect to their Intraclass Correlation Coefficient (ICC) where ICC was calculated from the granular data of the patients with MS. The numbers indicate the feature's labels as illustrated by the label in Table 2. Gray colors are features from the training set while green colors are the test set. Here we only include FC > 2 and ICC > 0.5. A diagram of all features that are statistically significant between HD and MS in the training and test set for patients with MS ICC is provided as Supplementary Figures S7, S8, respectively, in the Supplementary Material (HD and MS indicate healthy donors and multiple sclerosis patients, respectively).

**Figure 4:**
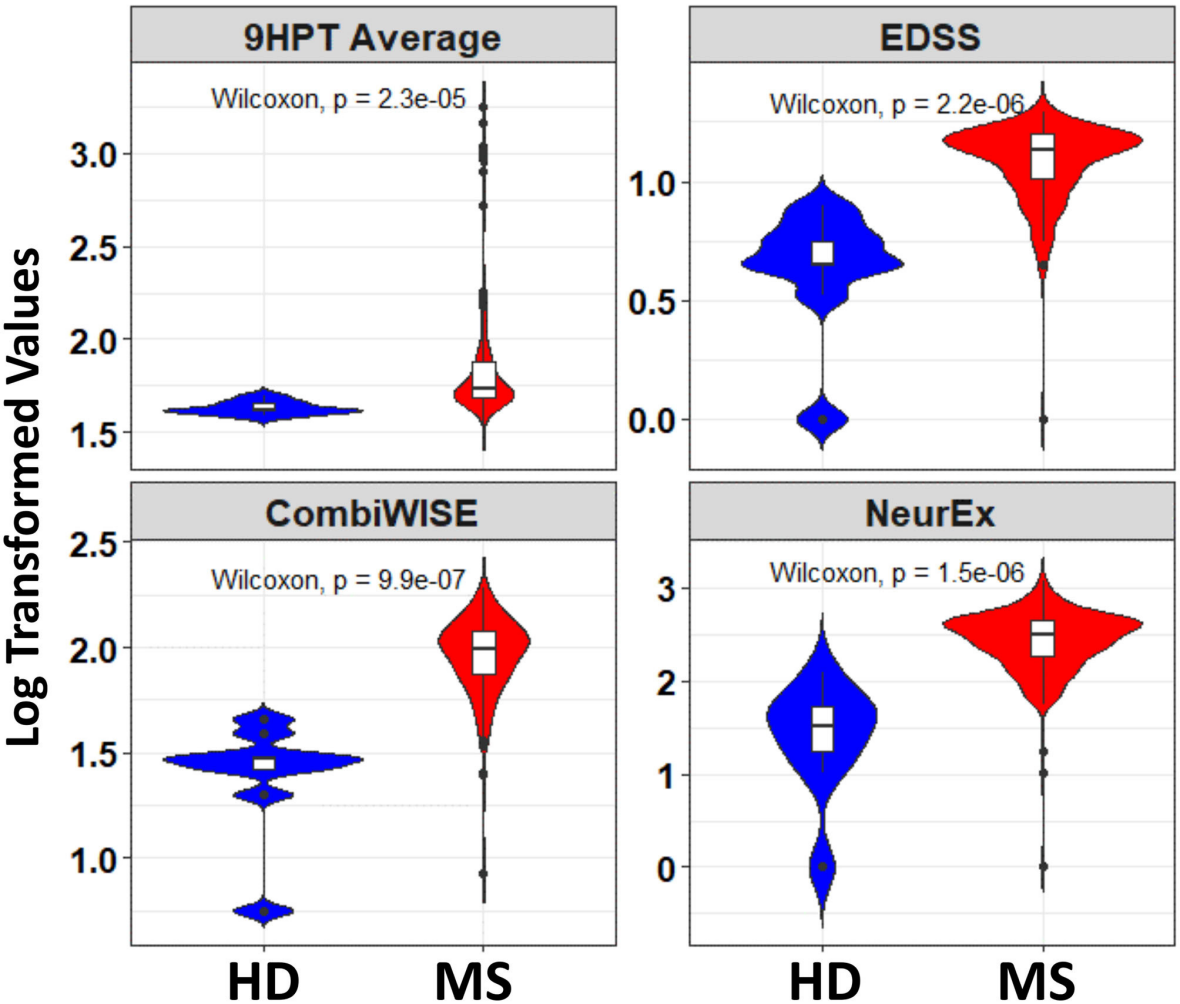
Violin and boxplot of selected clinical disability scales with respect to healthy donors and patients with MS. The blue color indicates the healthy donors while red shows patients with MS. There were multiple tests per subject in the MS group. Values are shown using a bi-symmetric log transformation (The p indicates the *p*-value of the unpaired Two-Samples Wilcoxon test for the mean comparison of HD and MS groups).

**Figure 5:**
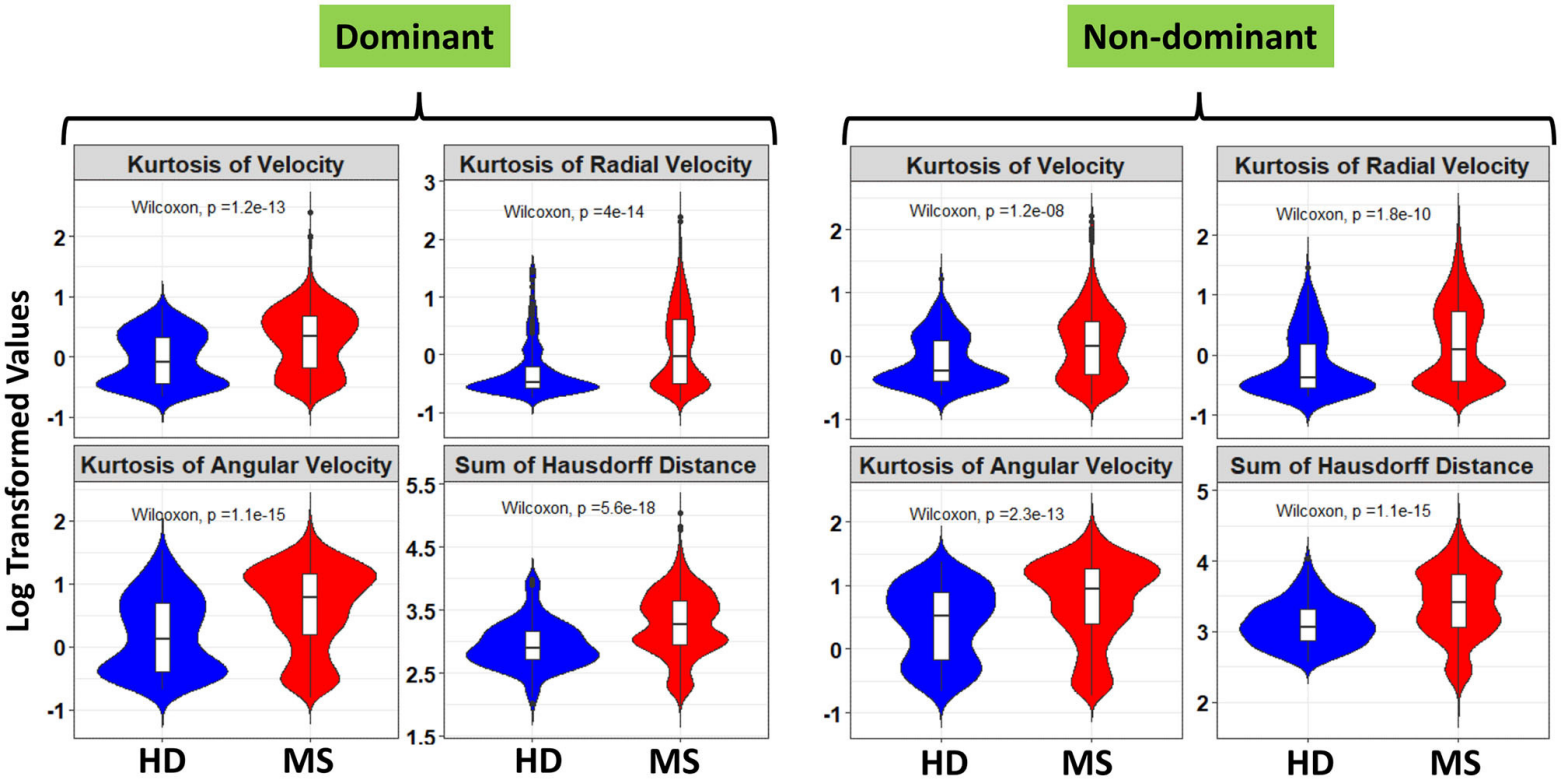
Violin and boxplot of the selected spiral-derived features with FC > 2 and ICC > 0.5 with respect to healthy donors and patients with MS at the difficulty level 1. The blue color indicates the healthy donors while red shows the patients with MS. There were multiple tests per subject in the HD and MS group. Values are shown using a bi-symmetric log transformation for both the dominant and non-dominant hand (The p indicates the *p*-value of the unpaired Two-Samples Wilcoxon test for the mean comparison of HD and MS groups).

**Figure 6:**
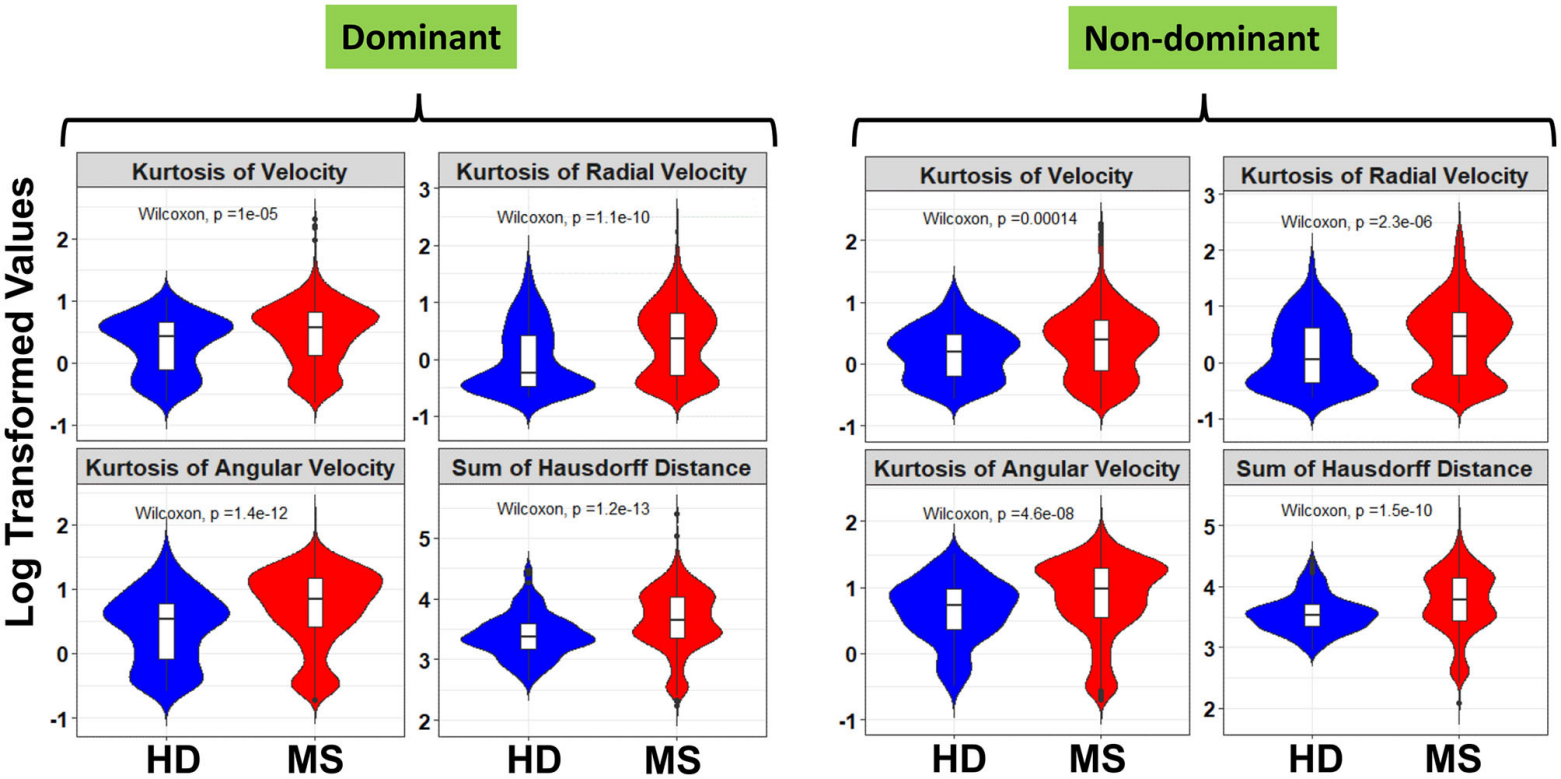
Violin and boxplot of the selected spiral-derived features with FC > 2 and ICC > 0.5 with respect to healthy donors and patients with MS at the difficulty level 2. The blue color indicates the healthy donors while red shows the patients with MS. There were multiple tests per subject in the HD and MS group. Values are shown using a bi-symmetric log transformation for both the dominant and non-dominant hand (The p indicates the *p*-value of the unpaired Two-Samples Wilcoxon test for the mean comparison of HD and MS groups).

**Figure 7:**
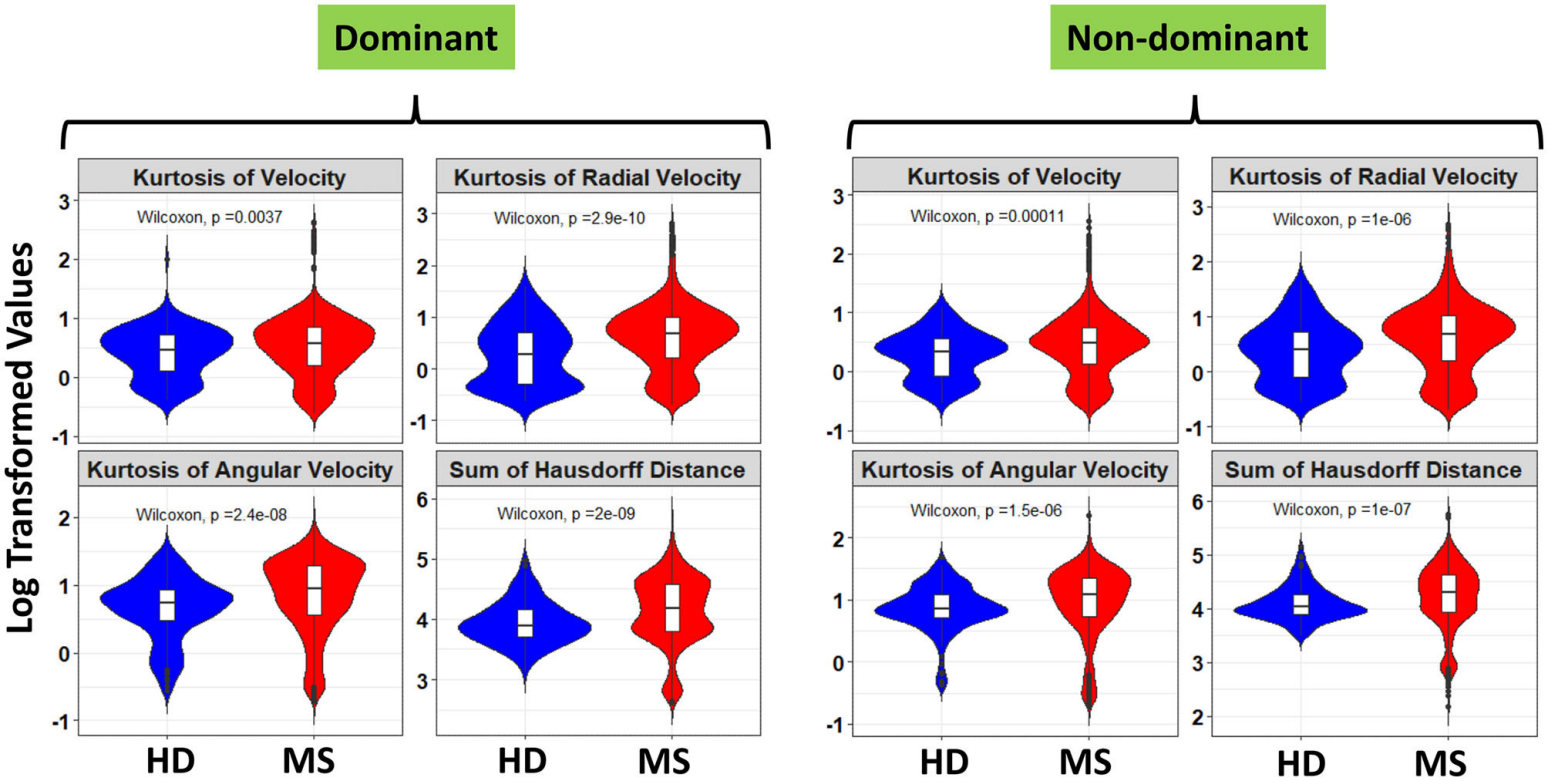
Violin and boxplot of the selected spiral-derived features with FC > 2 and ICC > 0.5 with respect to healthy donors and patients with MS at the difficulty level 3. The blue color indicates the healthy donors while red shows the patients with MS. There were multiple tests per subject in the HD and MS group. Values are shown using a bi-symmetric log transformation for both the dominant and non-dominant hand (The *p* indicates the *p*-value of the unpaired Two-Samples Wilcoxon test for the mean comparison of HD and MS groups).

**Figure 8:**
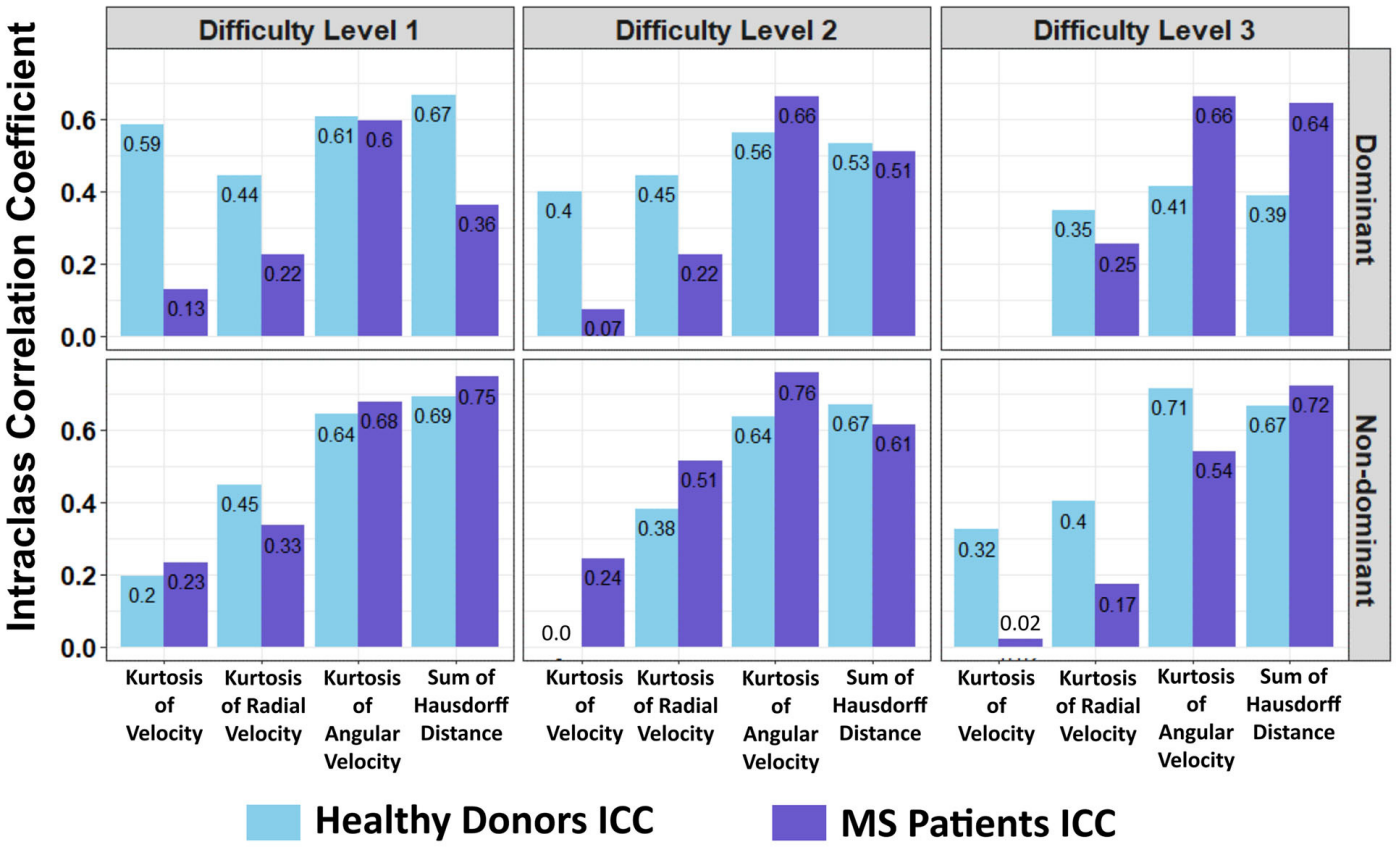
Comparison of the intraclass correlation coefficient (ICC) calculated from the granular data of the healthy vs. MS cohorts. The *x*-axis represents spiral-derived features with FC > 2 and ICC > 0.5 while the *y*-axis is the ICC (MS indicates multiple sclerosis patients).

Furthermore, a look at the FC of HD and MS with respect to the ICC indicated that only three spiral-derived features (kurtosis of velocity, kurtosis of angular velocity, and the sum of the Hausdorff distances) have FC > 2 and ICC > 0.5 when ICC was calculated using the granular data of HD (Figure 2). When ICC was computed for patients with MS, kurtosis of radial velocity, kurtosis of angular velocity, and the sum of Hausdorff distances have FC > 2 and ICC > 0.5 (Figure 3). In general, four spiral-derived features (i.e., kurtosis of velocity, kurtosis of radial velocity, kurtosis of angular velocity, and the sum of Hausdorff distances) were found to be statistically significant between HD and MS, have FC > 2, and have HD or MS patients ICC > 0.5. These features have a moderate strength of test-retest reliability and are significantly different in HD and MS as indicated by their FC and ICC (Figures 2, 3). There were statistically significant differences between HD and patients with MS in selected clinical features and the four most impactful spiral-derived features as depicted by violin and boxplots (see Figure 4 for boxplot of selected clinical features and Figures 5–7 for boxplots of the most impactful spiral-derived features at the difficulty level 1, 2, and 3, respectively). Most spiral-tracing features have a median value higher in the MS as compared to the HD (Figures 4–7). Also, the ICC of many patients with MS is also higher than that of HD (Figure 8). This is expected based on a higher inter-individual variance of spiral-tracing outcomes in MS vs. HD.

**Figure 9:**
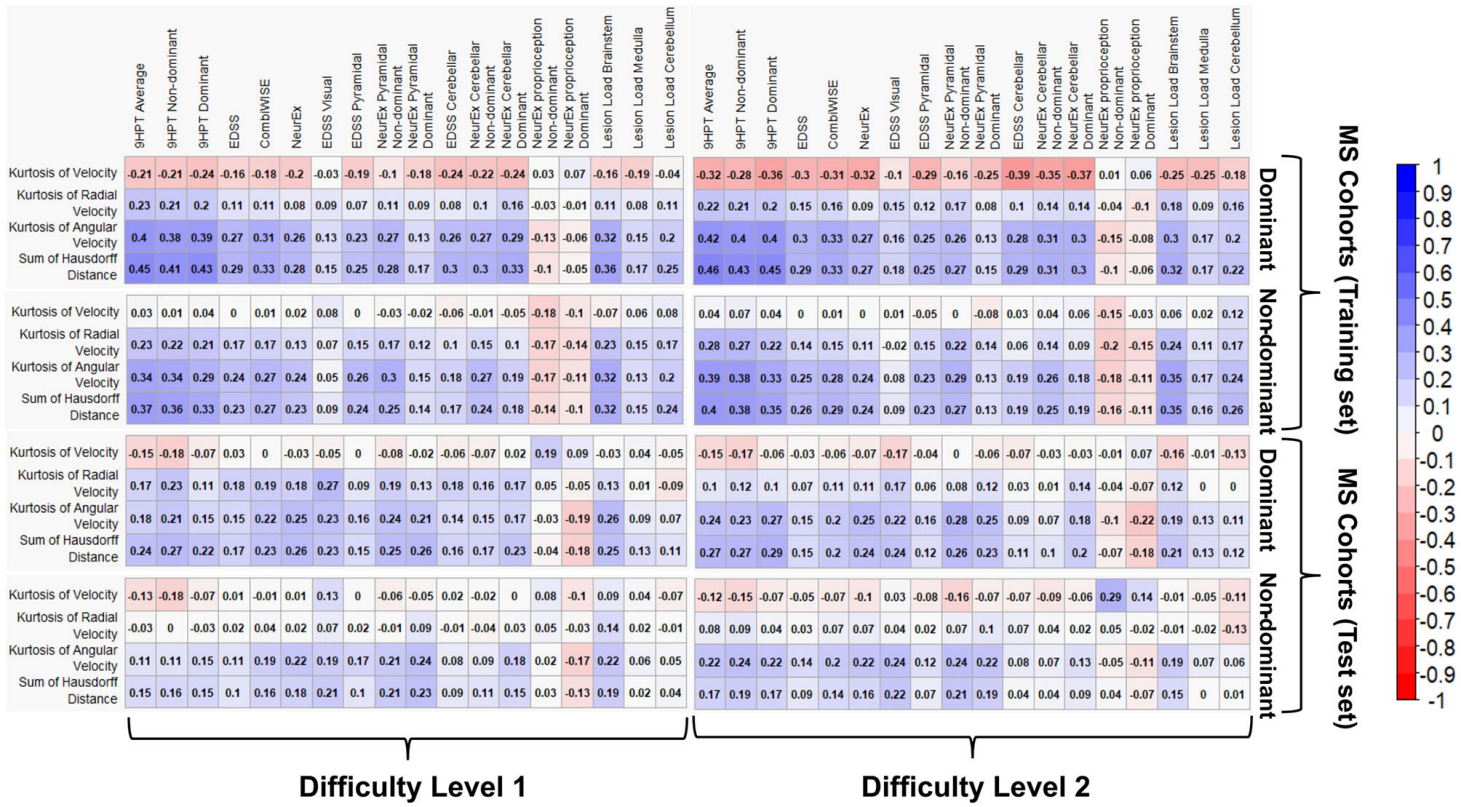
Spearman Rho correlation matrix between the statistically significant clinical disability and the top four most significant spiral-derived features based on FC at the difficulty level 1 and 2. The number indicates the Spearman correlation coefficient. Red is a negative correlation while blue stands for positive correlation. The white color indicates correlations that are not statistically significant at BH adjusted *p*-value of 0.05 (FC indicates Fold-Change, while BH denotes Benjamini–Hochberg).

To determine the relationship between the clinical disability scales and most impactful spiral-derived features (kurtosis of velocity, radial velocity, angular velocity, and the sum of Hausdorff distances), a Spearman Rho correlation matrix among the features was constructed (see Figure 9 for difficulty level 1 and 2, and Supplementary Figure S6 for difficulty level 3). In general, the highest correlations were seen among the 9HPT average, EDSS, CombiWISE, NeurEx^TM^, Lesion Load Brainstem, and the spiral-derived features at the dominant hand levels (Figure 9 and Supplementary Figure S6). Among the spiral-derived features, the sum of the Hausdorff distances had the highest correlations in both cohorts, but the strength of correlations was weak to moderate.

From the neurological examination subdomains, only motoric and cerebellar functions (but not proprioception) are reliably correlated with best spiral tracing features. We observed a very high positive correlation between the sum of Hausdorff distances and the time taken to complete the drawing in both the HD and MS patients (Supplementary Figures S7–S9; Spearman Rho > 0.97 for both dominant and non-dominant hand and at all difficulty levels). This is counterintuitive as we expected that increasing the speed of spiral drawing will negatively affect tracing accuracy. Instead, it appears that the disability and/or patient's confidence in his/her ability to trace the spiral affected both the speed and accuracy of the tracing congruently.

### ML Models of Best Spiral Tracing Features and Their Independent Cohort Validation

Four regression models (ElasticNet, SVR Radial, RF, and GBM) were used to evaluate the relationship between clinical disability scales and our four most impactful features (kurtosis of velocity, kurtosis of angular velocity, kurtosis of radial velocity, and the sum of the Hausdorff distances). The models had the best performance predicting the clinical disability scales in the (small; 22 subjects as presented in Table 1) HD cohort based on the mean RMSE and *R*^2^ across the 5-fold CV with 10 repetitions (see Supplementary Tables S2, S5, S8 for difficulty level 1, 2, and 3, respectively). Among the HD at the difficulty level 1, the ElasticNet model performed the best by explaining at most 85% in clinical disability scales when the dependent variables were CombiWISE or EDSS (Supplementary Table S2). When the dependent variables were 9HPT Average or NeurEx, SVR Radial had the best performance at the difficulty level 1 with an *R*^2^ value between 0.69 and 0.79 (Supplementary Table S2). The results of the percent variance explained in model outcomes at the difficulty levels 2 and 3 in HD were lower compared to the difficulty level 1 but still range between 30 and 76% (Supplementary Tables S5, S8).

Model's performance in the (much larger; 89 subjects as presented in Table 1) MS training cohort was much lower at all difficulty levels in comparison to results from the HD. The SVR Radial performed the best by explaining only 6–23% of the variance in clinical disability scale depending on the hand used (dominant or non-dominant), difficulty levels, and clinical disability scale (see Supplementary Tables S3, S6, S9 for difficulty level 1, 2, and 3, respectively).

However, the out-of-sample test performance (i.e., the independent validation cohort) were much lower compared to the result from the 5-fold CV of the training cohort (see *R*^2^ values between 0.0015 and 0.191 in Supplementary Tables S4, S7, S10 for difficulty level 1, 2, and 3, respectively). Of these, models with a 9HPT average yield the best performance with an R^2^ between 0.0304 and 0.1914 but only for the dominant hand (Supplementary Tables S4, S7, S10 for difficulty level 1, 2, and 3, respectively). However, in the independent test set, the GBM models generally validated the worst. Thus, we conclude that cross-validation of the training set is overly optimistic and does not reliably predict an independent test cohort performance.

**Figure 10:**
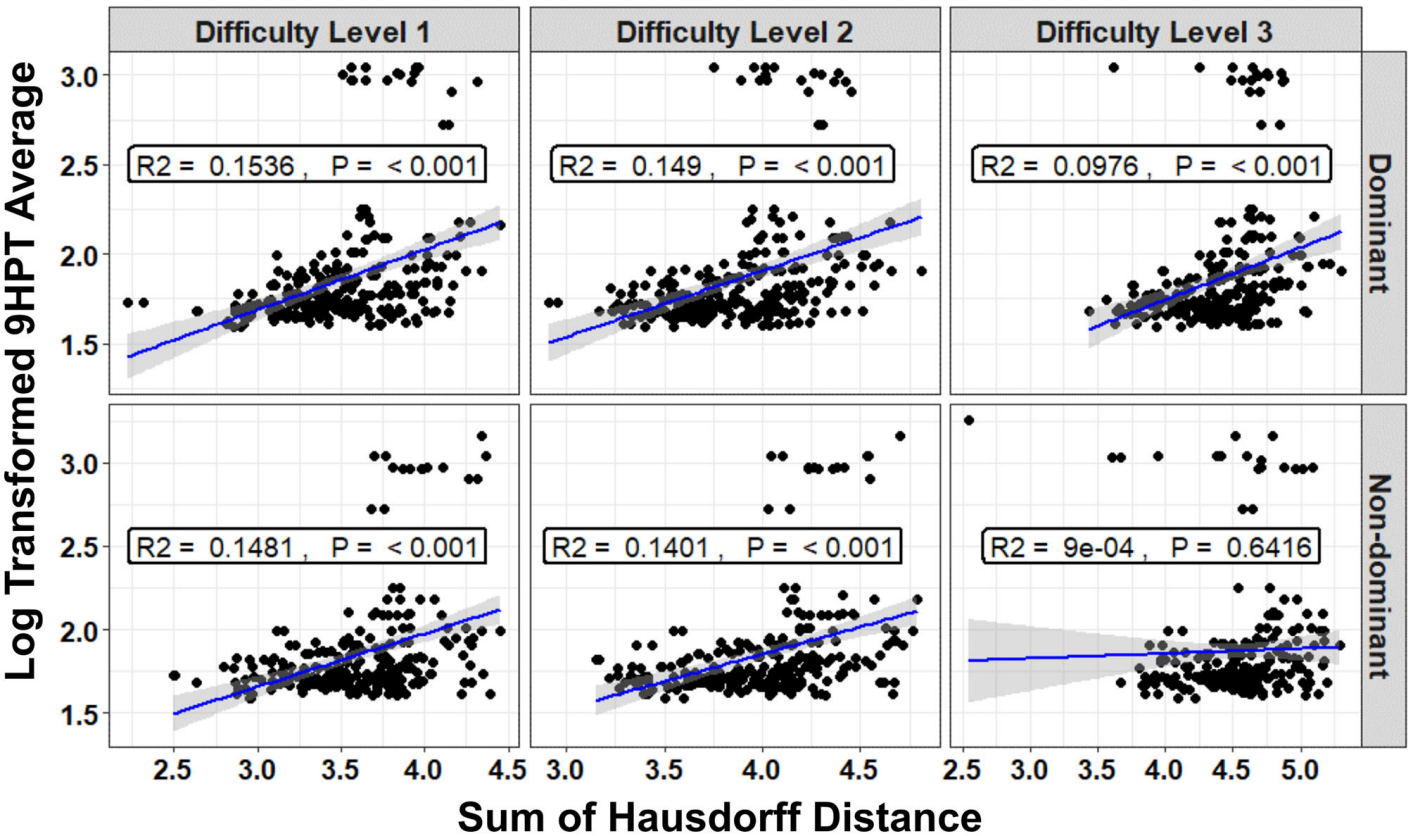
Relationship between the sum of the Hausdorff distances and the 9HPT average (in seconds) of the MS cohorts in black dots. Regression lines are shown in solid blue line while the gray shaded area constitutes the 95% confidence interval associated with the mean model's prediction (The R2 indicates the percent of the variance in the 9HPT average that can be explained by the sum of Hausdorff distances. P is the model's *p*-value).

Given that the sum of Hausdorff distances had the highest correlation with the clinical disability scale at all difficulty levels (Figure 9 and Supplementary Figure S6), linear regression models were constructed to measure the relationship between the disability scales and the sum of Hausdorff distances alone. In general, all clinical disability scales were positively correlated with the sum of Hausdorff distances (see Figure 10 for correlation with average 9HPT and Supplementary Figures S10–S12 for correlations with EDSS, CombiWISE, and NeurEx, respectively). The validation cohort performance of the sum of Hausdorff distances alone (Figure 10 for 9HPT) was comparable to the more complex ML-based models (i.e., *R*^2^ between 0.00834 and 0.1593 in Supplementary Table S11).

**Figure 11:**
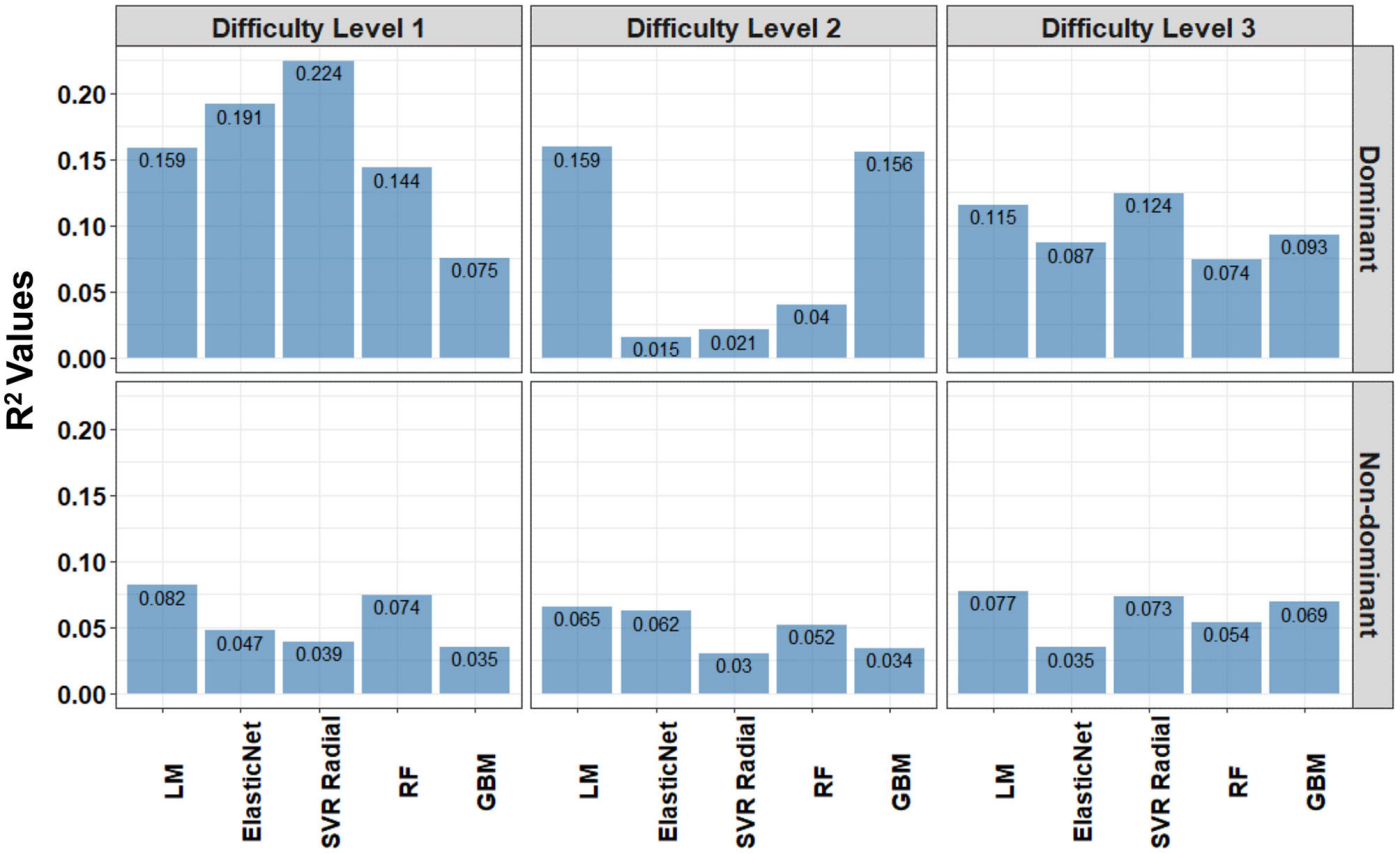
The out-of-sample test performance (i.e., independent validation) of the predictive regression models when 9HPT average (in seconds) is the response variable and the sum of Hausdorff distances is the only explanatory variable in the linear regression model (LM). The explanatory variables in the Elastic Net (ElasticNet), Support Vector Regression with Radial Basis Function kernel (SVR Radial), Random Forest (RF), and Stochastic Gradient Boosting (GBM) models are Kurtosis of Velocity, Kurtosis of Radial Velocity, Kurtosis of Angular Velocity, and the sum of Hausdorff distances. The test performance was measured using R^2^ of model predictions per dominant and non-dominant hands among the patients with MS at the difficulty level 1, 2, and 3.

Overall, we observed better predictive accuracy (based on R^2^) in the dominant hand category than the non-dominant hand category and for difficulty levels 1 and 2 compared to difficulty level 3 in the dominant hand (Figure 11). These results remained consistent when controlling for the age and gender variables in all the models (see Supplementary Tables S12, S13 for cross-validation and out-of-sample test performance of the ML models at the difficulty level 1 for MS subjects, respectively). Similar results were found from the CV of the training cohort in Creagh et al. (9), where the authors observed that the mean absolute error (MAE) was higher in non-dominant hand models than dominant models for HD subjects. However, it was observed that non-dominant hand models more accurately predicted 9HPT than dominant hand regression models in the MS subjects (9).

## Discussion

Test reproducibility (measured as ICC, signal-to-noise ratio, or concordance coefficient) is generally given lesser importance in the test design than test sensitivity. Presented analyses of the Spiral tracing support the notion that test reproducibility is an essential determinant of its clinical utility. Achieving high reproducibility of digital tests should be at forefront of the medical app developers.

Spiral tracing is a complex test that includes many neurological functions: fine finger movements, negatively affected by motoric dysfunction, proprioceptive loss and cerebellar dysfunction, eye–hand coordination, affected by vision, oculomotor and cerebellar dysfunctions, and cognition or anxiety associated with anticipated test difficulty. Additionally, the precision of the test is affected by the time of test execution, even though we observed, counterintuitively, a strong *positive* correlation between measures of test accuracy such as the sum of Hausdorff distances and the time it took to perform the tracing, even in HD. This suggests that time was not the primary driver of the inaccuracy of tracing. Rather, combined neurological disability and/or lack of confidence in the ability to perform tests, both fast and accurately, negatively affected the test performance. This explains why Spiral tracing features that adjusted the accuracy of tracing for the velocity performed worse than the most successful accuracy measure, the sum of Hausdorff distances.

The comparison of cross-validation performance of the MS training cohort with the performance of the ML-based models in the true independent validation cohort demonstrated that training cohort cross-validation performance overestimates performance of the test in subjects who did not contribute to the model development: when the performance of the strongest ML-based models (i.e., modeling 9HPT) are compared between cross-validation of the training cohort and the independent validation cohort, all four ML algorithms greatly overestimated the performance of the models in the independent validation cohort. The best feature of the Spiral tracing (the sum of Hausdorff distances) performed comparatively to the ML-based models in the independent validation (Figure 8). This overestimation of the model performance from the training cohort data, even when training cohort results are based on cross-validation, is the rule we observed uniformly in the past decade of our experience with independent validation of complex models. We used to not even show the training cohort results in our publications, as we consider them irrelevant. However, after realizing that the vast majority of ML studies in biomedical literature do not use independent validation cohort and that most readers and reviewers consider cross-validation of the training cohort equivalent to the independent validation, we now routinely publish training cohort data to demonstrate the level of overfitting in comparison to the truly independent validation cohort.

We also point out that the cross-validation performance of the training cohort does not faithfully predict even the ranking of the models.

In this regard, because the COVID-19 pandemic precluded us from recruiting an independent validation cohort of HD, we consider the cross-validation performance of the HD models unrealistically optimistic (especially because of the small number of HD) and fully expect that those models represent overfitting. Therefore, the HD models should not be considered promising without independent validation.

The poor performance of these ML-based models was, in our experience, expected based on poor ICCs and weak univariate correlations of individual Spiral tracing features with the gold standard of neurological disability measures and brain MRI markers of CNS injury in MS cohorts. In comparison, much simpler tests such as rapidly tapping on the screen of the smartphone correlated much stronger with analogous disability measures (i.e., up to Spearman Rho of 0.76) and were also much more intra-individually stable (7). Interestingly, both 9HPT and smartphone finger tapping differentiated MS from HD better for non-dominant hand; the observation was reproduced for 9HPT in multiple studies (4, 7, 19). We interpreted this observation by functional repair: even though MS likely affects both hands equally, the daily use of the dominant hand promotes repair, both as remyelination and the establishment of new synaptic circuits by remaining neurons. Therefore, digital tests of the non-dominant hand, which has less rehabilitation/repair, are more sensitive to measuring the difference between patients with MS and HD and to measuring the progression of disability in time. Surprisingly, the non-dominant hand performed much worse in the Spiral tracing test, in both MS cohorts. We believe that this was due to higher intra-individual variance/greater noise, which has little to do with disability and more to do with test complexity. As test complexity increased to Level 3, the reproducibility and clinical relevance of the Spiral tracing features decreased quite dramatically.

We recognize that poor reproducibility of the Spiral tracing observed in our study may be mitigated in situations where spiral tracing is performed on tablets and therefore, the spiral is much larger (10). We developed NeuFun-TS for smartphones rather than tablets, due to the larger worldwide prevalence and greater availability of different sensors in the former compared to the latter. The test selection must consider the screen size difference for apps targeting different mobile devices.

In conclusion, in self-administered digital measurements of neurological functions, the designers should strive to develop tests that are easy to perform and therefore highly reproducible, but still reflect a specific neurological (dys)function. These simpler tests will likely be (by design) less sensitive than tests that depend on multiple neurological functions, but the sensitivity can be restored by aggregating results from multiple simple tests, as is being done in NeuFun-TS. However, the total time necessary to complete all tests in NeuFun-TS will likely determine compliance with longitudinal testing. Therefore, as Spiral tracing does not add clinical value beyond existing tapping (19), balloon popping (7), and level tests (18), we plan to drop Spiral tracing from NeuFun-TS standard tests. Spiral tracing Fourier analysis to identify tremor, frequency, and severity may still be very useful in patients with movement disorders.

## Data Availability Statement

The datasets presented in this study can be found in online repositories. The name of the repository and accession number can be found at: GitHub, https://github.com/bielekovaLab/Bielekova-Lab-Code/tree/master/FormerLabMembers/Messan_Komi.

## Ethics Statement

The studies involving human participants were reviewed and approved by National Institute of Allergy and Infectious Diseases (NIAID) scientific review and the National Institutes of Health (NIH) Institutional Review Board. The patients/participants provided their written informed consent to participate in this study.

## Author Contributions

BB conceived the study design. BB, LP, TH, YK, VM, and PK performed the app construction and clinical data collection. BB and KM conceived the analytical methodologies. KM performed the statistical analysis and ML modeling with their respective visualizations. All authors participated in writing and editing the manuscript.

## Funding

This research was supported by the Intramural Research Program of the National Institutes of Health (NIH) and the National Institute of Allergy and Infectious Diseases (NIAID). KM was supported in part by an appointment to the NIAID Research Participation Program. This program is administered by the Oak Ridge Institute for Science and Education through an interagency agreement between the U.S. Department of Energy (DOE) and the National Institute of Allergy and Infectious Diseases (NIAID). ORISE is managed by ORAU under DOE contract number DE-SC0014664.

## Author Disclaimer

All opinions expressed in this paper are the author's and do not necessarily reflect the policies and views of NIAID, DOE, or ORAU/ORISE.

## Conflict of Interest

The authors declare that the research was conducted in the absence of any commercial or financial relationships that could be construed as a potential conflict of interest.

## Publisher's Note

All claims expressed in this article are solely those of the authors and do not necessarily represent those of their affiliated organizations, or those of the publisher, the editors and the reviewers. Any product that may be evaluated in this article, or claim that may be made by its manufacturer, is not guaranteed or endorsed by the publisher.
