# Supplementary material for: Assessment of Smartphone-Based Spiral Tracing in Multiple Sclerosis Reveals Intra-Individual Reproducibility as a Major Determinant of the Clinical Utility of the Digital Test
Source: Front Med Technol. 2022 Feb 1;3:714682. doi: 10.3389/fmedt.2021.714682 (PMC8844508; doi:10.3389/fmedt.2021.714682)
Supplement: Supplementary file 1 [file Data_Sheet_1.docx]

Supplementary Material

# Supplementary Figures and Tables

## Supplementary Figures


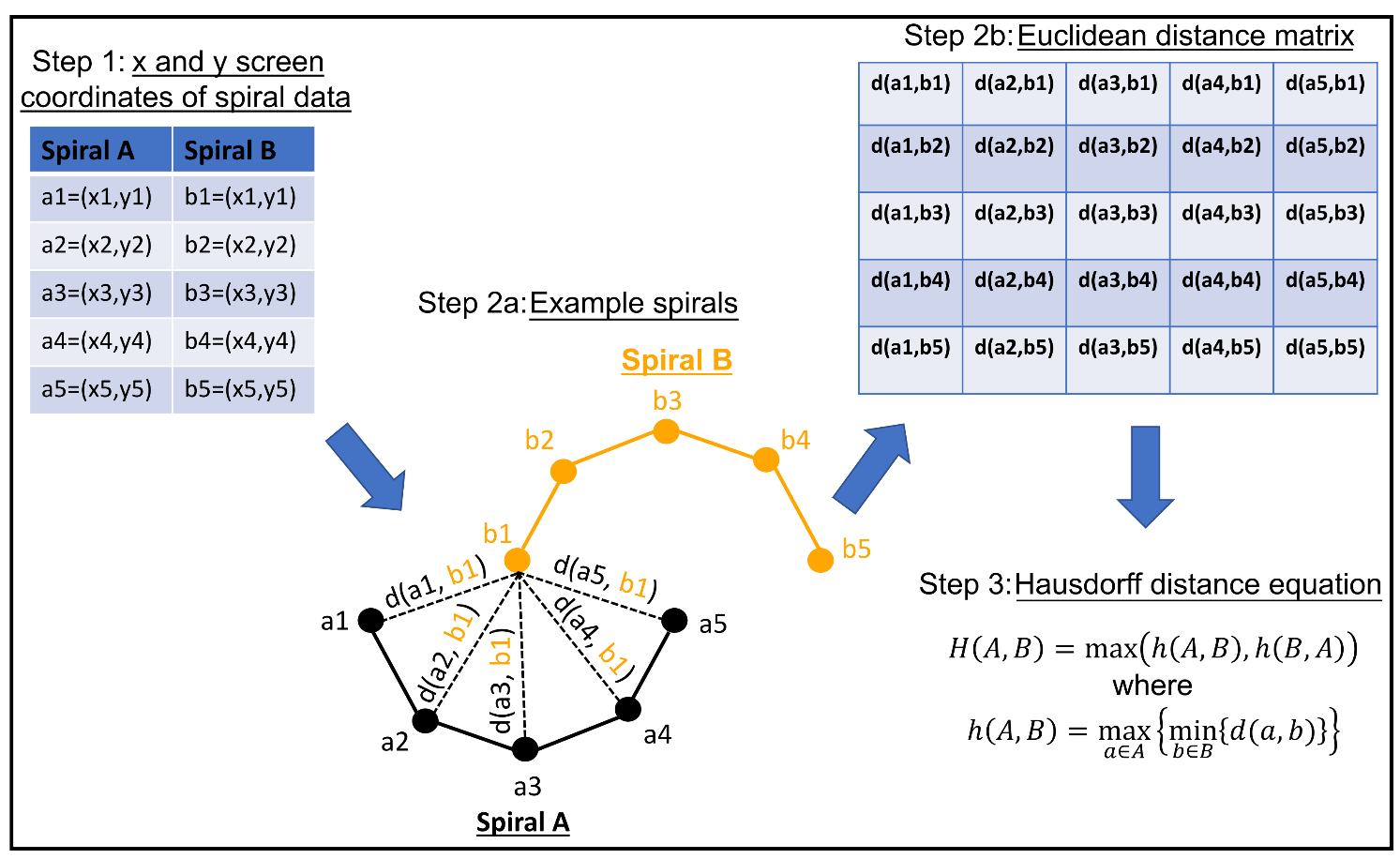


Supplementary Figure 1. An example of the different steps to calculate the Hausdorff distance between two spiral curves. Step 1 represents an example of x and y coordinates reference spiral data (in orange; Spiral B) and cohort’s drawing data (in black; Spiral A). Step 2a is the Euclidean distance from b1 to each point in Spiral A denoted as d(ai, b1), i=1,2,3, 4, and 5. Dashed lines indicate the Euclidean distances. Step 2b represents the distance matrix from each point in Spiral B to each point in Spiral A. Step 3 finally provides the equation to calculate the Hausdorff distance after obtaining the distance matrix. A more detail explanation on the computation of Hausdorff distance can be found in (Dubuisson & Jain, 1994; Huttenlocher, Klanderman, & Rucklidge, 1993).


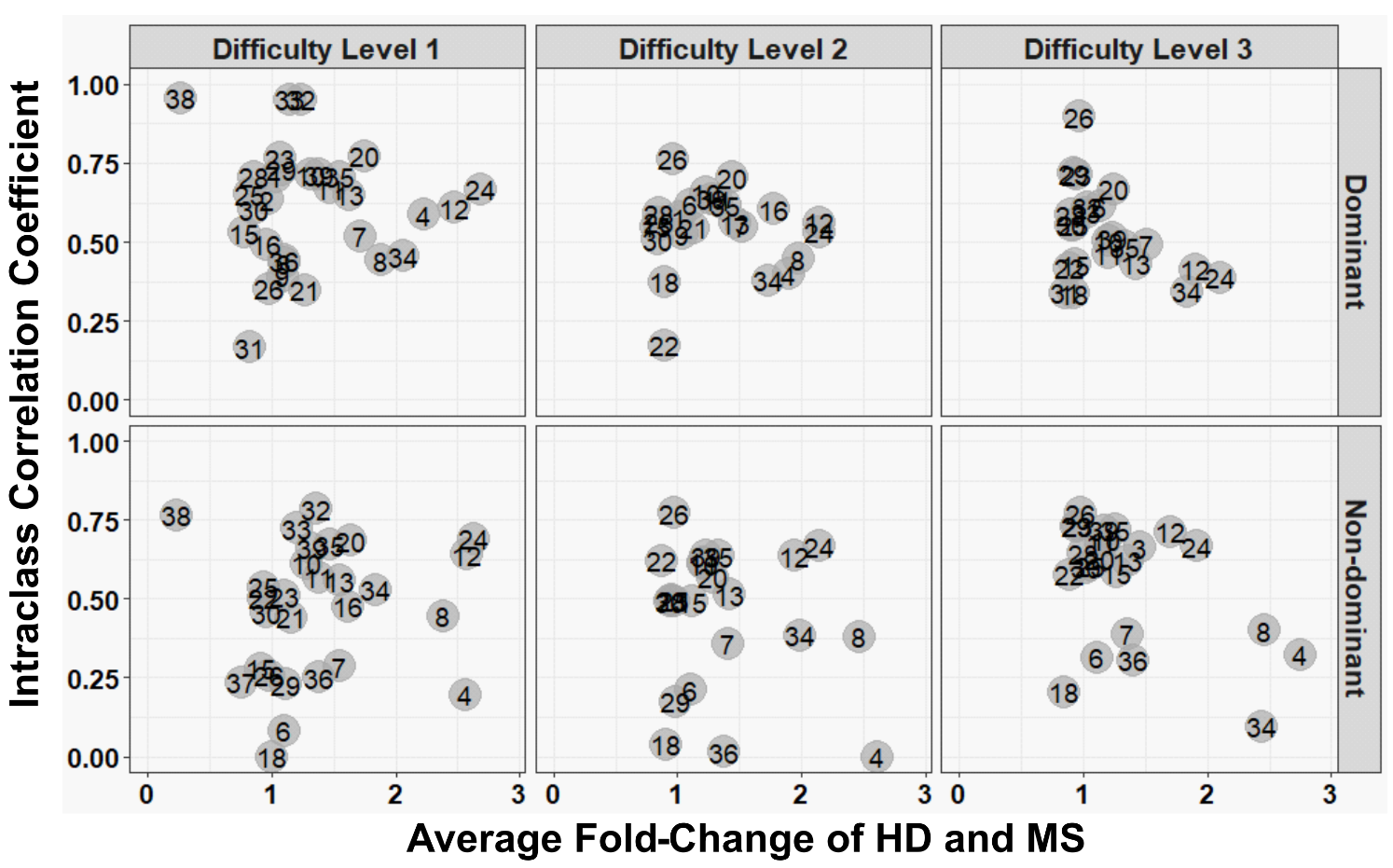


Supplementary Figure 2. Average fold change of healthy donors (HD) and multiple sclerosis (MS) patients of the spiral derived features with respect to their interclass correlation coefficient (ICC) from the training set. ICC was calculated from the granular data of the HD. The numbers indicate the feature’s labels as illustrated by the label in Table 1.


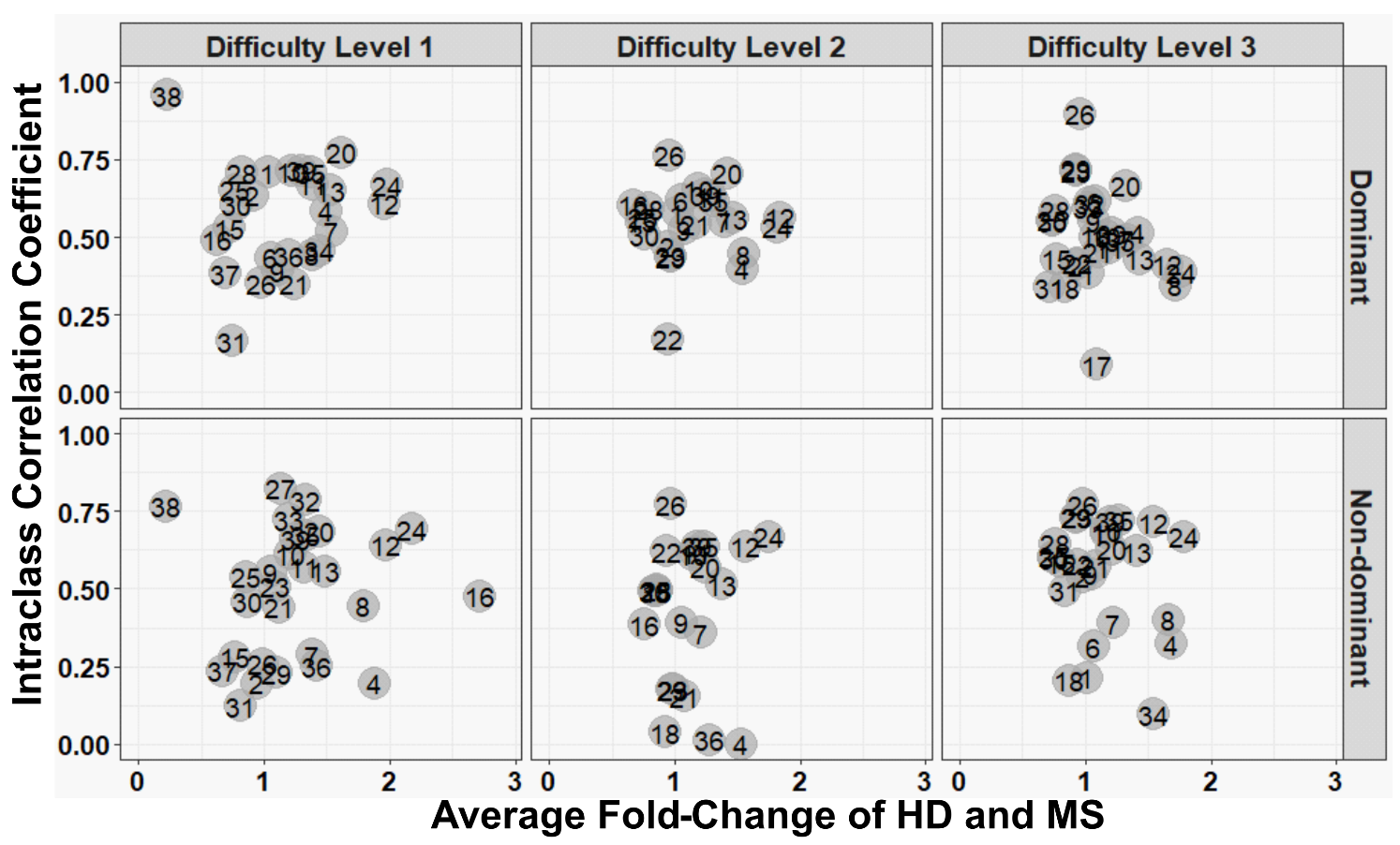


Supplementary Figure 3. Average fold-change of healthy donors (HD) and multiple sclerosis (MS) patients of the spiral derived features with respect to their interclass correlation coefficient (ICC) from the test set. ICC was calculated from the granular data of the HD. The numbers illustrate the feature’s labels as indicated by the label in Table 1.


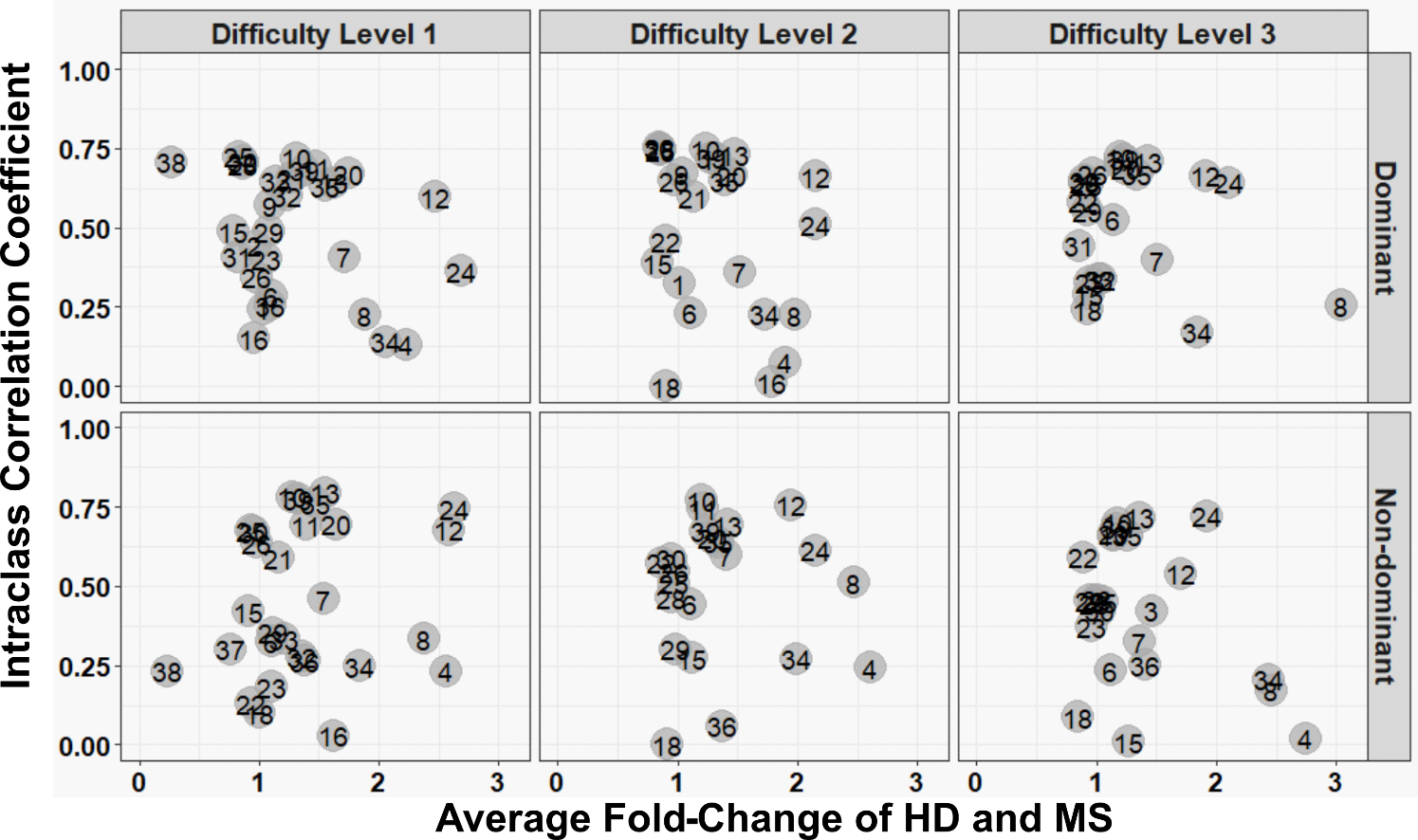


Supplementary Figure 4. Average fold-change of healthy donors (HD) and multiple sclerosis (MS) patients of the spiral derived features with respect to their interclass correlation coefficient (ICC) from the training set. ICC was calculated from the granular data of the MS patients. The numbers indicate the feature’s labels as illustrated by the label in Table 1.


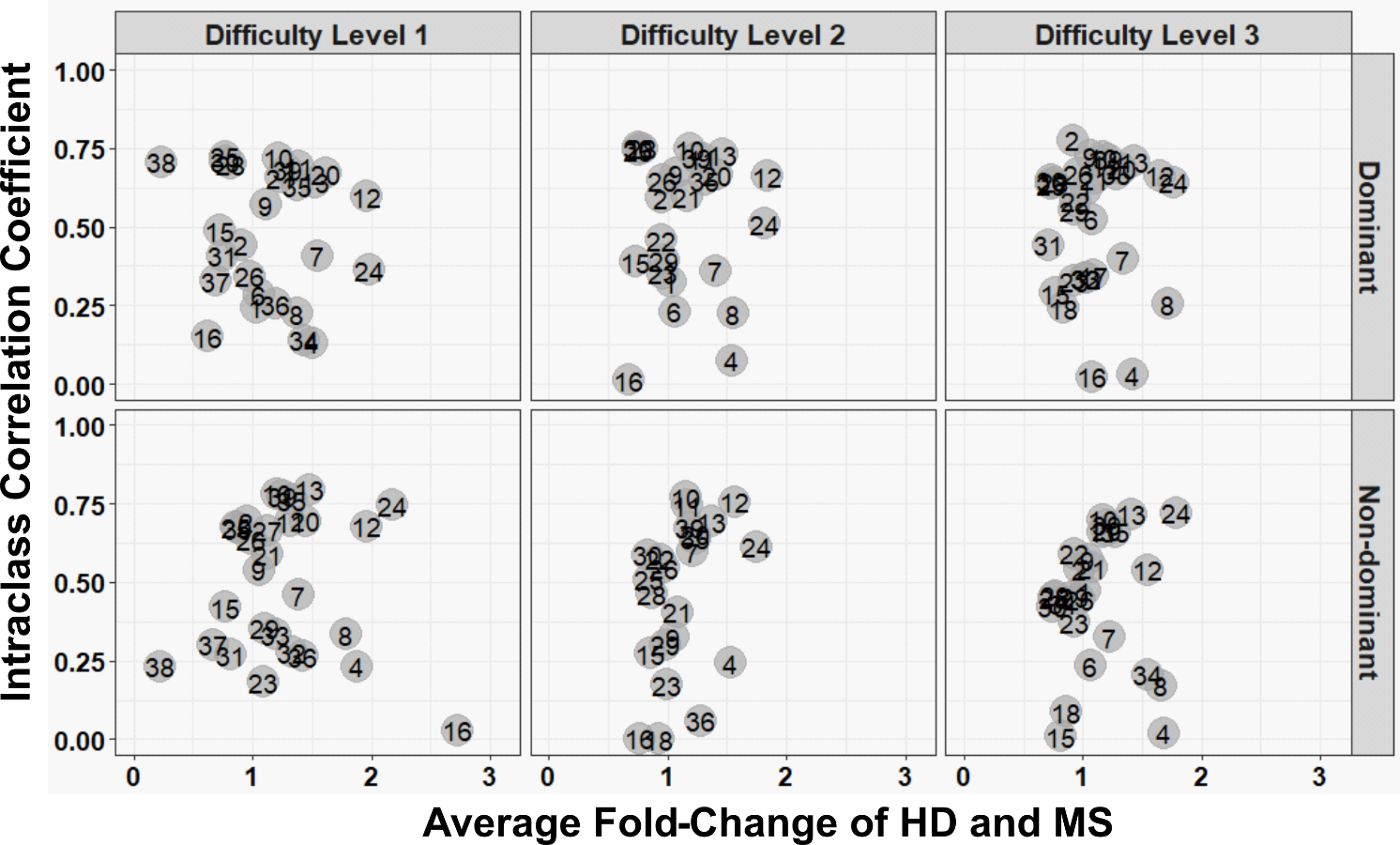


Supplementary Figure 5. Average fold-change of healthy donors and multiple sclerosis (MS) patients of the spiral derived features with respect to their interclass correlation coefficient (ICC) from the test set. ICC was calculated from the granular data of the MS patients. The numbers illustrate the feature’s labels as indicated by the label in Table 1.


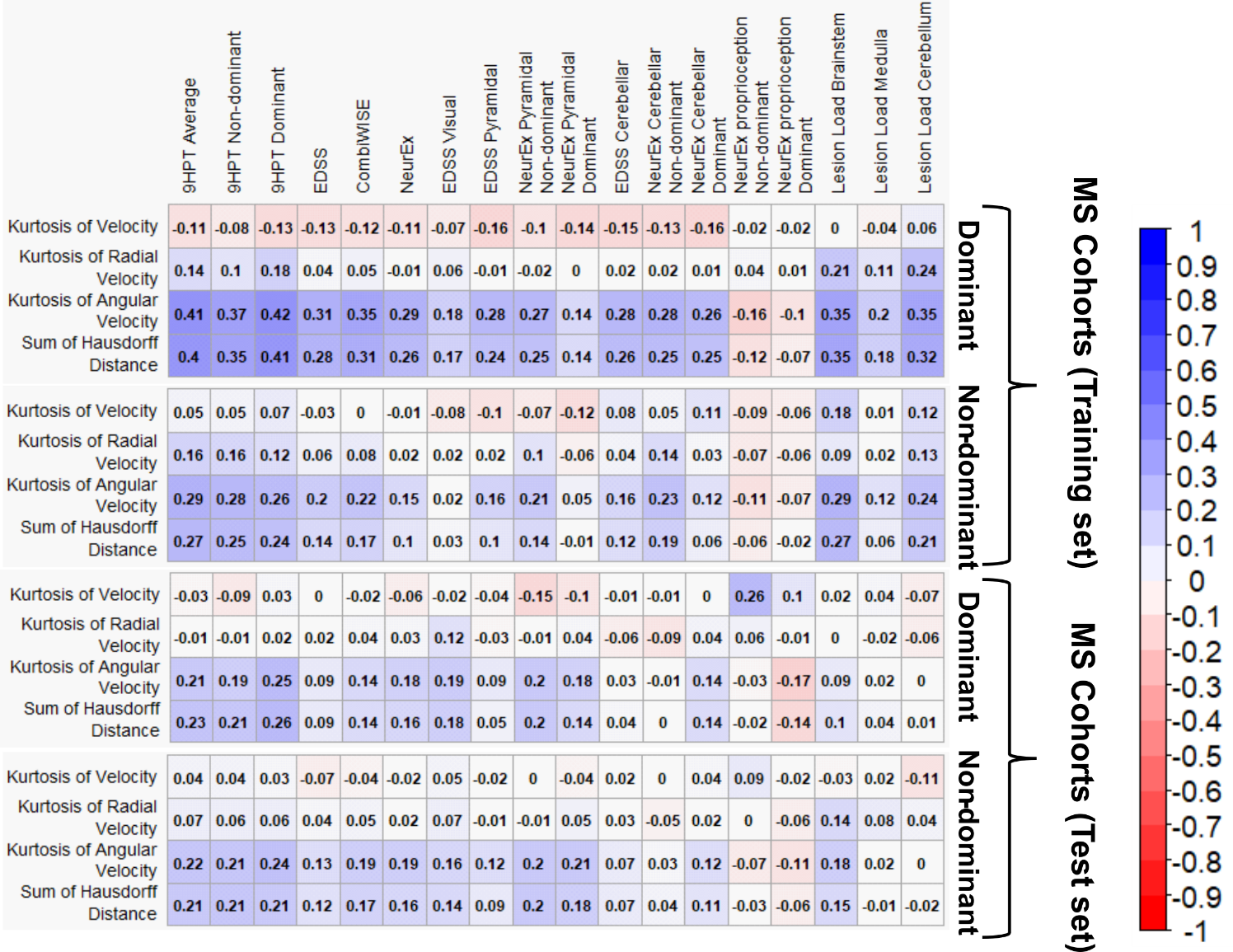


Supplementary Figure 6. Spearman Rho correlation matrix between the statistically significant clinical disability and the top four most significant spiral derived features based on fold-change at the difficulty level 3. The number indicates the Spearman correlation coefficient. Red is negative correlation while blue stand for positive correlation. The white color indicates correlation that are not statistically significant at Benjamini-Hochberg adjusted p-value of 0.05.


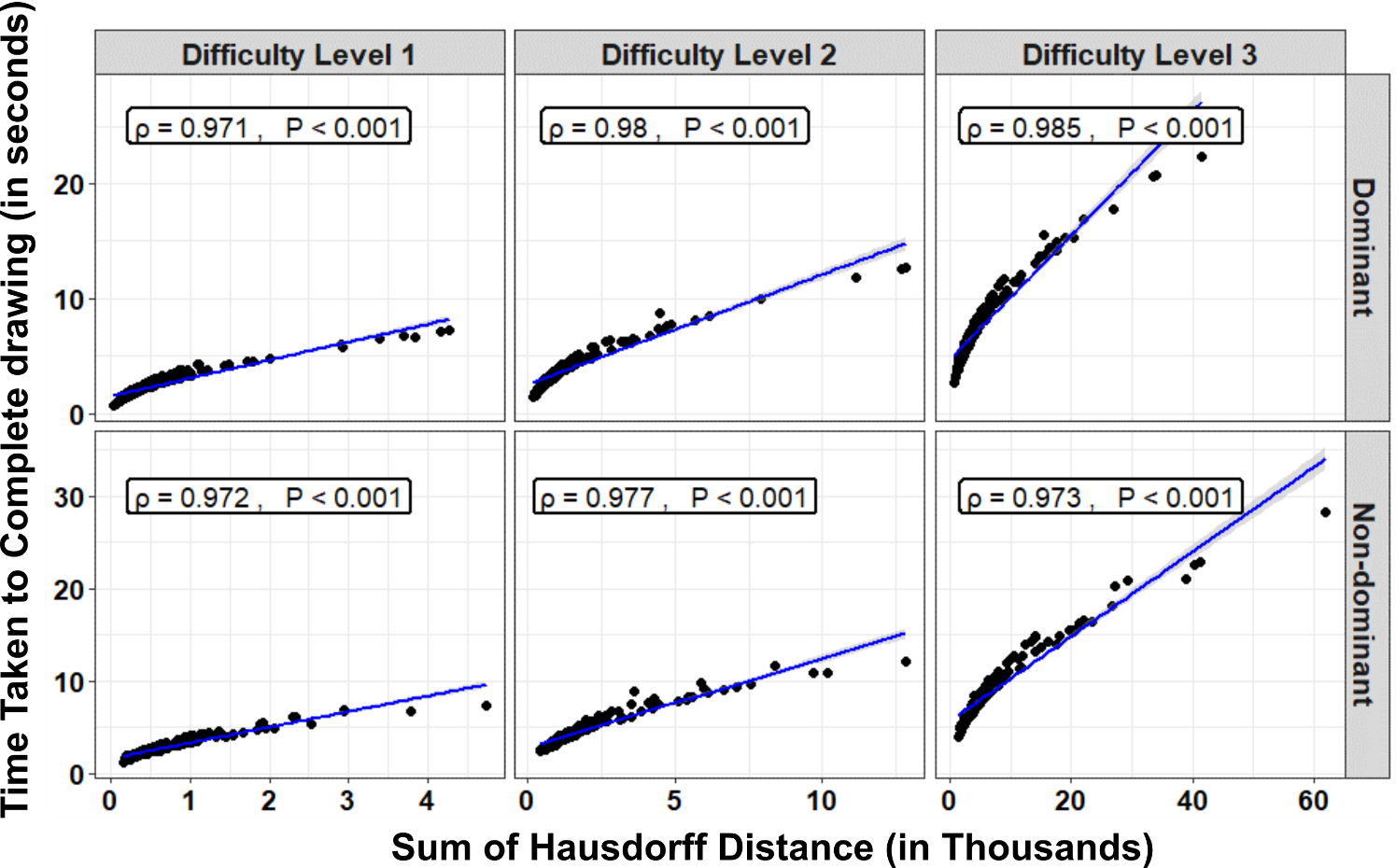


**Supplementary Figure 7.** Relationship between the sum of the Hausdorff distances and the time taken to complete the spiral drawing among the healthy donors (shown in black dots). Regression lines are shown in solid blue line. The ρ is the Spearman correlation coefficient between the variables while P is the p-value of the correlation.


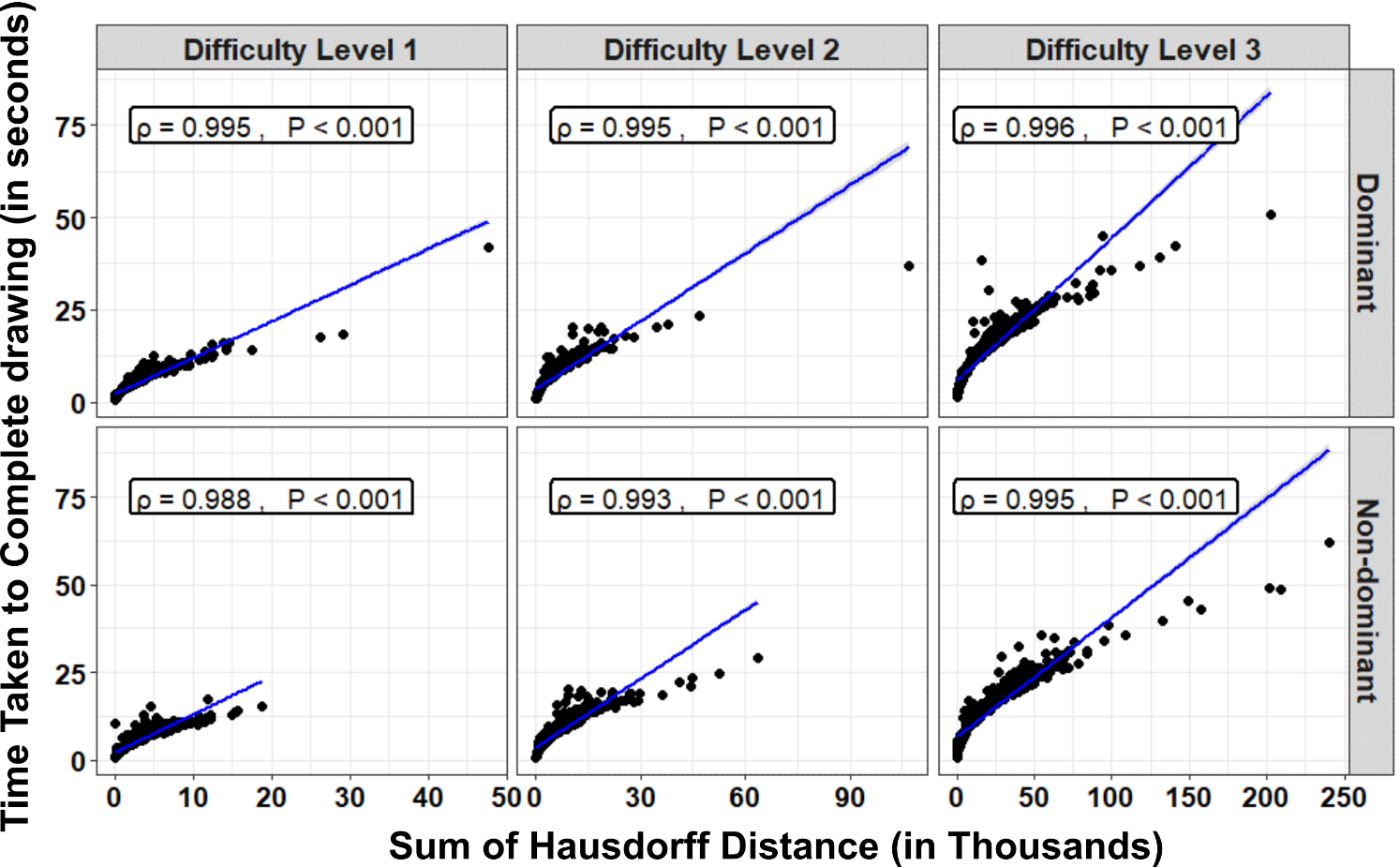


**Supplementary Figure 8**. Relationship between the sum of the Hausdorff distances and the time taken to complete the spiral drawing among the multiple sclerosis patients – training set (shown in black dots). Regression lines are shown in solid blue line. The ρ is the Spearman correlation coefficient between the variables while P is the p-value of the correlation.


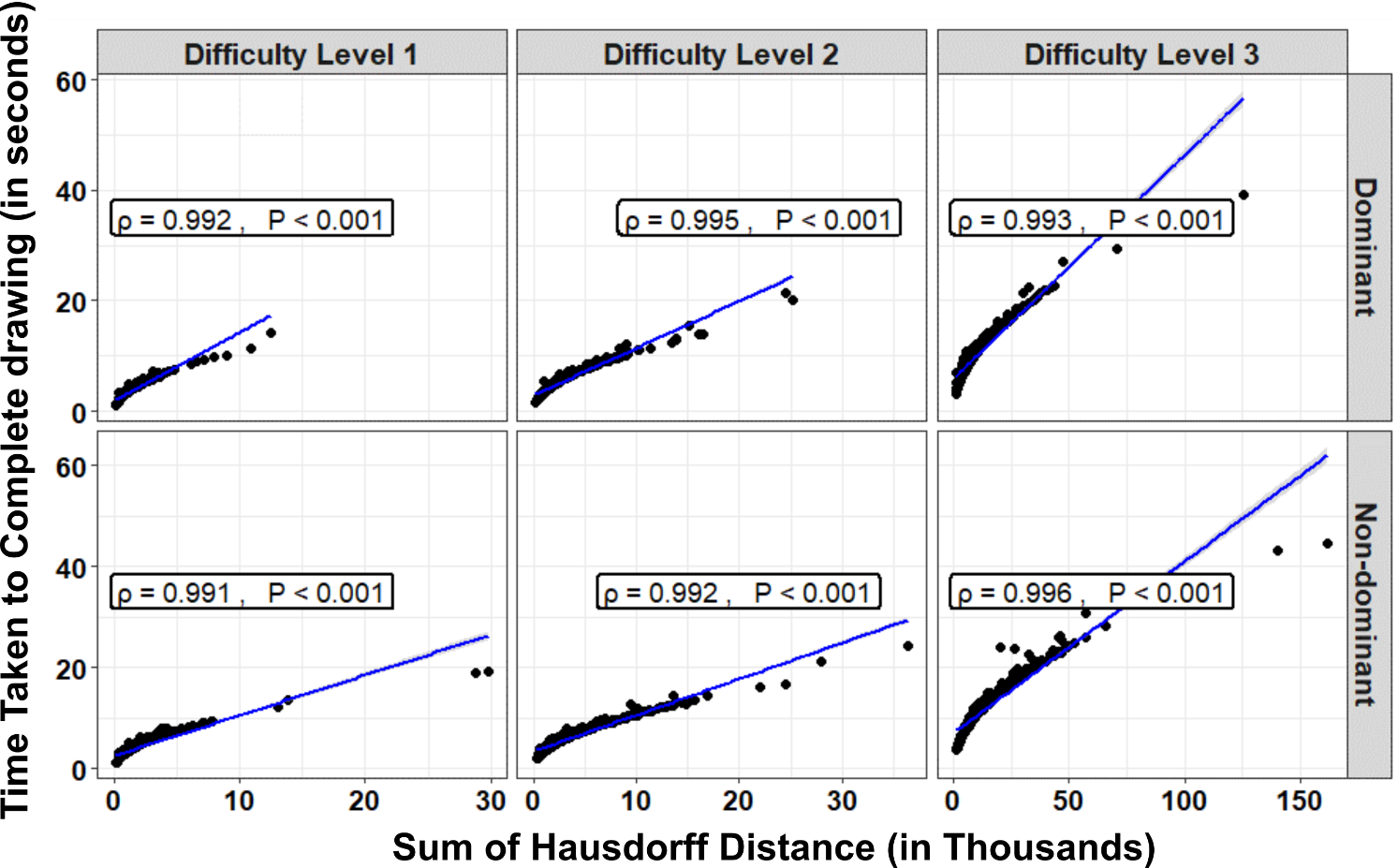


Supplementary Figure 9. Relationship between the sum of the Hausdorff distances and the time taken to complete the spiral drawing among the multiple sclerosis patients – test set (shown in black dots). Regression lines are shown in solid blue line. The ρ is the Spearman correlation coefficient between the variables while P is the p-value of the correlation.


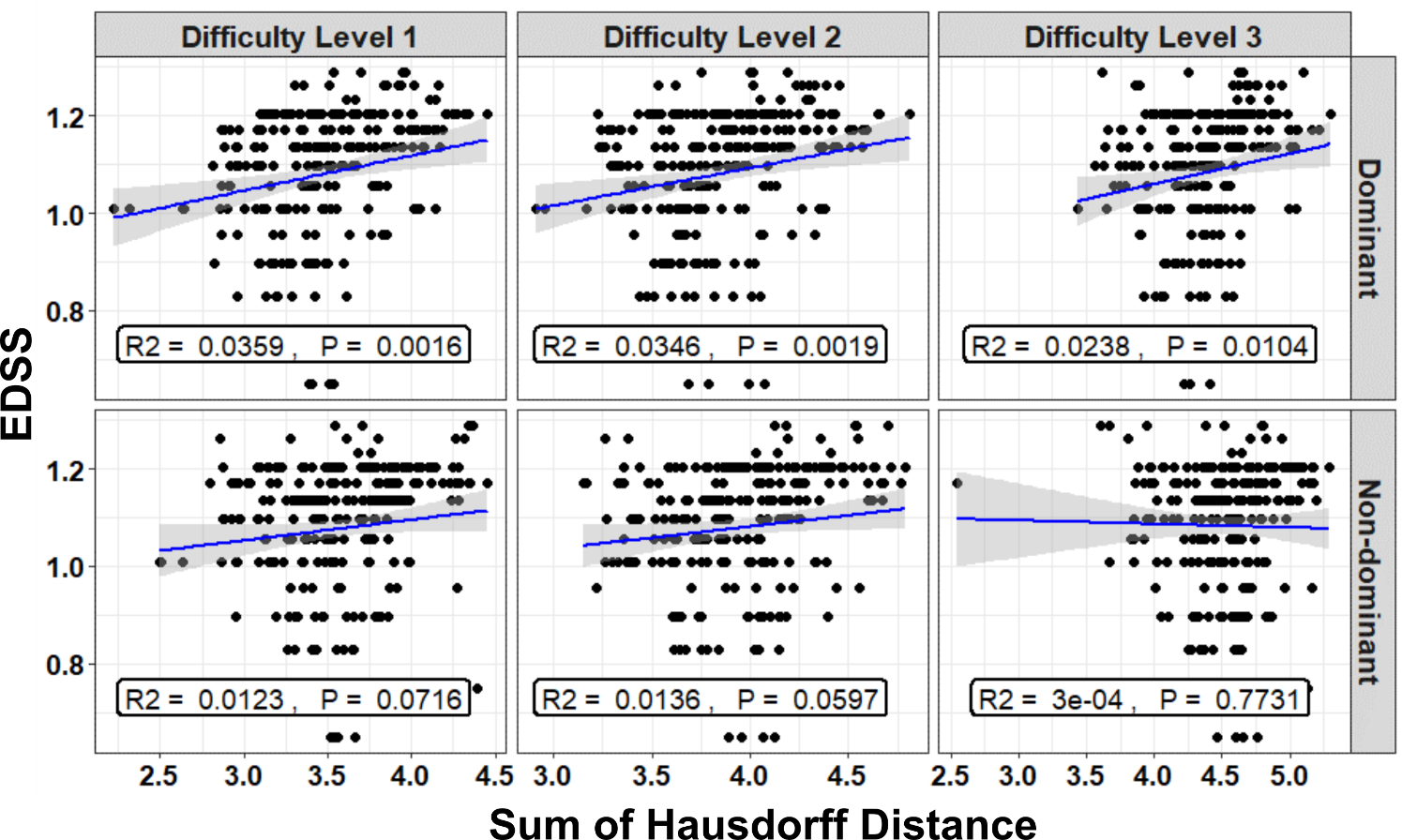


Supplementary Figure 10. Relationship between the sum of the Hausdorff distances and EDSS of the multiple sclerosis patients in black dots. Linear regression lines are shown in solid blue line while the gray shaded area constitute the 95% confidence interval associated with the mean model’s prediction. The R2 indicates the percent of variance in EDSS that can be explained by the sum of Hausdorff distances. P is the model’s p-value.


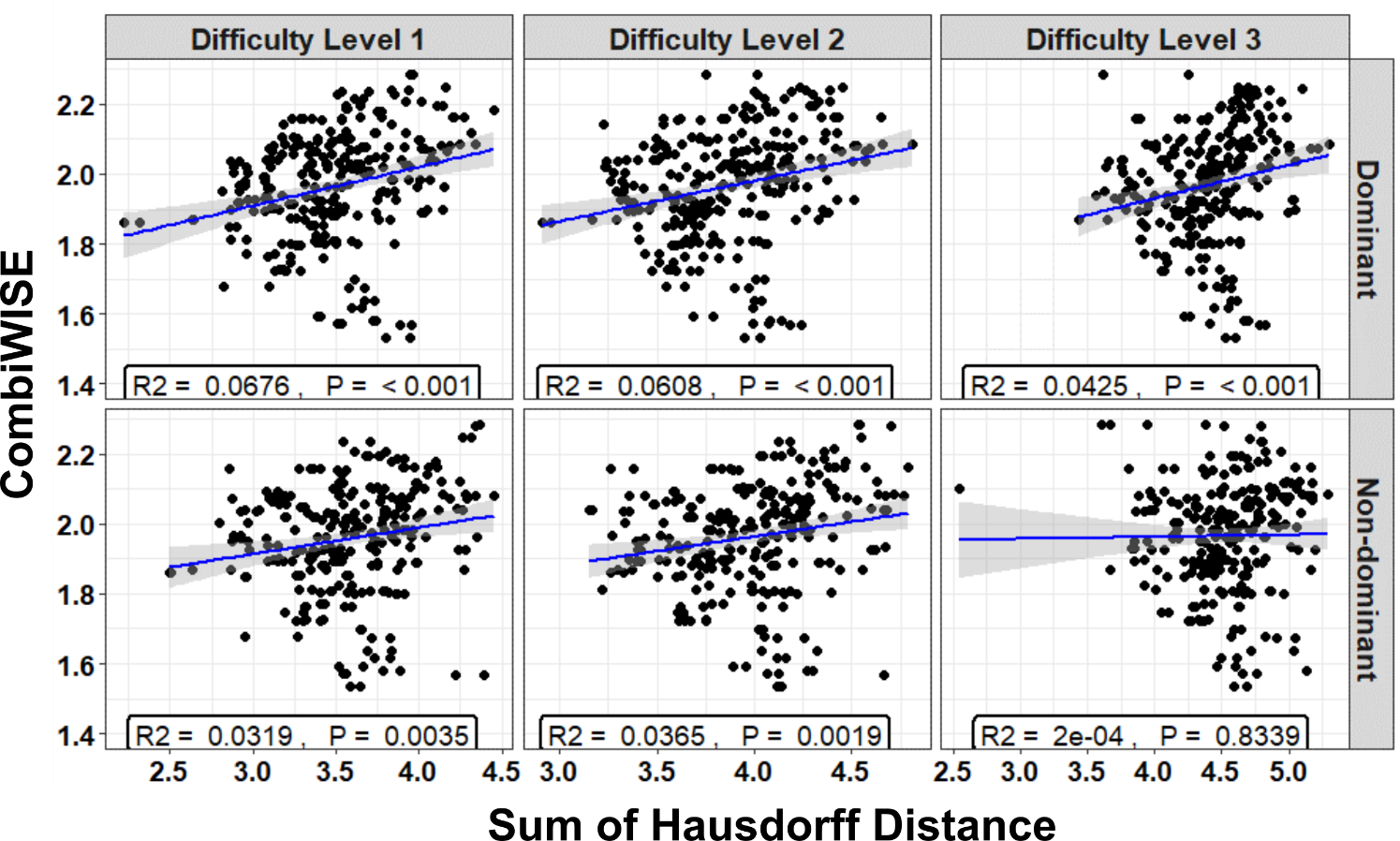


Supplementary Figure 11. Relationship between the sum of the Hausdorff distances and CombiWISE of the multiple sclerosis patients in black dots. Linear regression lines are shown in solid blue line while the gray shaded area constitute the 95% confidence interval associated with the mean model’s prediction. The R2 indicates the percent of variance in CombiWISE that can be explained by the sum of Hausdorff distances. P is the model’s p-value.


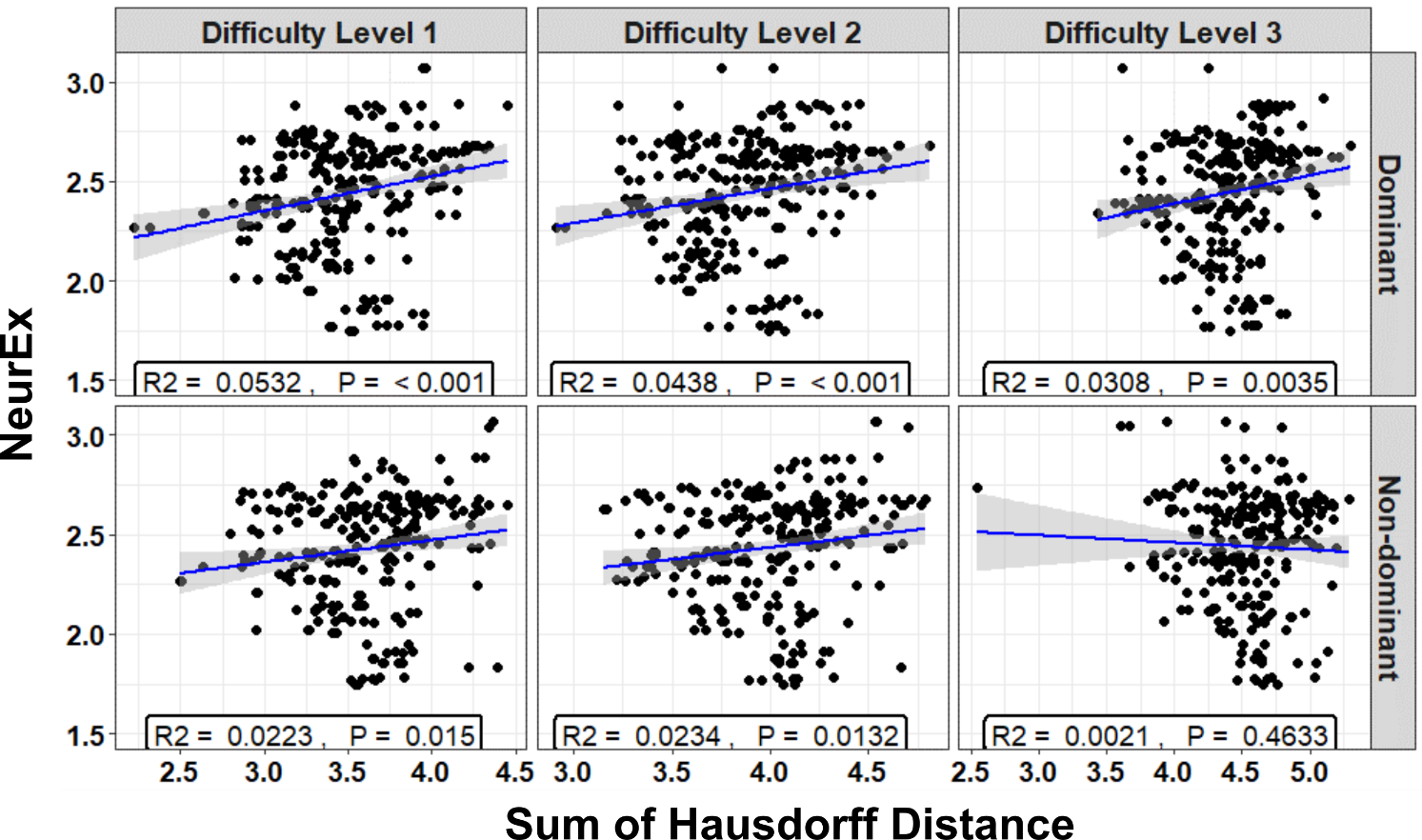


Supplementary Figure 12. Relationship between the sum of the Hausdorff distances and NeurEx of the multiple sclerosis patients in black dots. Linear regression lines are shown in solid blue line while the gray shaded area constitute the 95% confidence interval associated with the mean model’s prediction. The R2 indicates the percent of variance in NeurEx that can be explained by the sum of Hausdorff distances. P is the model’s p-value.

## Supplementary Tables

Supplementary Table 1. List of features relating to upper-extremity function calculated from the spiral drawing test. When applicable, references are provided for each calculated feature. Dashed symbols indicate not applicable.

| **Label** | **Description** | **References** | **Mean** | **Standard Deviation** |
| --- | --- | --- | --- | --- |
| F1 | Sum of velocity | - | 114.325 | 42.865 |
| F2 | Coefficient of variation of velocity | - | 0.395 | 0.165 |
| F3 | Skewness of velocity | - | -0.413 | 1.040 |
| F4 | Kurtosis of velocity | - | 1.622 | 6.166 |
| F5 | Sum of radial velocity | - | 23.510 | 11.284 |
| F6 | Coefficient of variation of radial velocity | - | 0.862 | 0.219 |
| F7 | Skewness of radial velocity | - | 1.234 | 1.013 |
| F8 | Kurtosis of radial velocity | - | 2.652 | 10.073 |
| F9 | Sum of angular velocity | - | 275.333 | 116.626 |
| F10 | Coefficient of variation of angular velocity | - | 1.334 | 0.463 |
| F11 | Skewness of angular velocity | - | 2.183 | 1.077 |
| F12 | Kurtosis of angular velocity | - | 4.552 | 5.220 |
| F13 | Sum of estimated pressure | - | 48.346 | 35.638 |
| F14 | Maximum Power Spectral Density (PSD) of velocity | (Creagh et al., 2020; Erasmus et al., 2001) | 2.308 | 3.457 |
| F15 | Dominant Frequency of velocity | (Creagh et al., 2020) | 0.253 | 0.260 |
| F16 | Maximum PSD of radial velocity | (Creagh et al., 2020; Erasmus et al., 2001) | 0.225 | 2.710 |
| F17 | Dominant Frequency of radial velocity | (Creagh et al., 2020) | 0.792 | 1.032 |
| F18 | Maximum PSD of angular velocity | (Creagh et al., 2020; Erasmus et al., 2001) | 21.856 | 31.710 |
| F19 | Dominant Frequency of angular velocity | (Creagh et al., 2020) | 1.652 | 1.478 |
| F20 | Approximate Entropy of velocity | (Creagh et al., 2020; Mevludin Memedi, Aghanavesi, & Westin, 2016; M. Memedi et al., 2015) | 0.306 | 0.187 |
| F21 | Approximate Entropy of radial velocity | (Creagh et al., 2020) | 0.388 | 0.156 |
| F22 | Approximate Entropy of angular velocity | (Creagh et al., 2020) | 0.250 | 0.070 |
| F23 | Maximum Hausdorff Distance (HDis) | (Creagh et al., 2020; Dubuisson & Jain, 1994; Huttenlocher et al., 1993) | 2.984 | 0.368 |
| F24 | Sum of HDis | (Creagh et al., 2020) | 5863.503 | 10862.331 |
| F25 | Sum of HDis divided by time taken to complete drawing | (Creagh et al., 2020) | 0.706 | 0.537 |
| F26 | Interquartile Range of Sum of HDis | (Creagh et al., 2020) | 0.604 | 0.037 |
| F27 | Sum of HDis normalized by touchpoints at the beginning | (Creagh et al., 2020) | 0.259 | 0.212 |
| F28 | Sum of HDis normalized by touchpoints at the end | (Creagh et al., 2020) | 0.258 | 0.165 |
| F29 | Sum of HDis in the middle 15-85% | (Creagh et al., 2020) | 2.875 | 0.367 |
| F30 | Sum of HDis in the middle 15-85% normalized by time taken to complete drawing | (Creagh et al., 2020) | 0.675 | 0.503 |
| F31 | Error calculated using Area Under the Curve | (Creagh et al., 2020) | 0.264 | 0.195 |
| F32 | Mean Square Error | (Asamoah, Ofori, Opoku, & Danso, 2018; Creagh et al., 2020) | 0.630 | 0.099 |
| F33 | Root Mean Square Error | (Creagh et al., 2020) | 0.790 | 0.074 |
| F34 | Center of Shoot | (Creagh et al., 2020) | 0.141 | 0.227 |
| F35 | Time taken to complete drawing | - | 6.827 | 5.182 |
| F36 | Total Asymmetry of patient drawing | (Creagh et al., 2020) | 0.176 | 0.111 |
| F37 | True Asymmetry in comparison with reference shape | - | 0.074 | 0.075 |
| F38 | 2D image Correlation between two images | (Aljanabi, Hussain, & Lu, 2018; Creagh et al., 2020) | 0.007 | 0.131 |
| F39 | Image entropy of shape drawn | (Creagh et al., 2020; Tsai, Lee, & Matsuyama, 2008) | 0.109 | 0.061 |
| F40 | Image entropy of shape drawn with respect to reference shape | (Creagh et al., 2020) | 0.998 | 0.008 |

Supplementary Table 2. Root Mean Square Error (RMSE) and R^2^ of model predictions of the clinical disability scales per dominant and non-dominant hands among the healthy donors at the difficulty level 1. Models are build using 5-fold cross-validation (CV) with 10 repetitions. Results are provided using mean ± SD where the mean and SD are the mean and Standard Deviation across CV repetitions. ElasticNet, SVR Radial, RF, and GBM represent respectively the Elastic net, Support Vector Regression with Radial Basis Function kernel, Random Forest, and Stochastic Gradient Boosting regression models. NA indicates cannot be computed.

| **Outcome variable** | **Models** | **Dominant hand** | | **Non-dominant hand** | |
| --- | --- | --- | --- | --- | --- |
|  |  | **RMSE** | **R^2^** | **RMSE** | **R^2^** |
| **9HPT Average (C1)** | **ElasticNet** | 0.03 ± 0.01 | 0.61 ± 0.40 | 0.03 ± 0.01 | 0.48 ± 0.36 |
|  | **SVR Radial** | 0.03 ± 0.01 | 0.69 ± 0.37 | 0.03 ± 0.01 | 0.58 ± 0.33 |
|  | **RF** | 0.03 ± 0.01 | 0.71 ± 0.34 | 0.03 ± 0.01 | 0.55 ± 0.33 |
|  | **GBM** | 0.03 ± 0.01 | 0.62 ± 0.37 | 0.03 ± 0.01 | 0.58 ± 0.35 |
| **EDSS (C4)** | **ElasticNet** | 0.06 ± 0.02 | 0.80 ± 0.25 | 0.22 ± 0.11 | 0.42 ± 0.30 |
|  | **SVR Radial** | 0.07 ± 0.02 | 0.68 ± 0.35 | 0.22 ± 0.14 | 0.31 ± 0.34 |
|  | **RF** | 0.07 ± 0.02 | 0.72 ± 0.33 | 0.18 ± 0.08 | 0.45 ± 0.36 |
|  | **GBM** | 0.07 ± 0.03 | 0.77 ± 0.27 | 0.25 ± 0.12 | 0.37 ± 0.34 |
| **CombiWISE (C5)** | **ElasticNet** | 0.05 ± 0.02 | 0.85 ± 0.21 | 0.21 ± 0.11 | 0.58 ± 0.33 |
|  | **SVR Radial** | 0.06 ± 0.02 | 0.84 ± 0.23 | 0.20 ± 0.07 | 0.53 ± 0.36 |
|  | **RF** | 0.06 ± 0.02 | 0.83 ± 0.27 | 0.18 ± 0.09 | 0.52 ± 0.36 |
|  | **GBM** | 0.05 ± 0.02 | 0.84 ± 0.27 | 0.24 ± 0.13 | 0.47 ± 0.38 |
| **NeurEx (C6)** | **ElasticNet** | 0.30 ± 0.08 | 0.61 ± 0.35 | 0.51 ± 0.17 | 0.42 ± 0.31 |
|  | **SVR Radial** | 0.21 ± 0.05 | 0.79 ± 0.28 | 0.51 ± 0.22 | 0.43 ± 0.33 |
|  | **RF** | 0.28 ± 0.07 | 0.70 ± 0.36 | 0.40 ± 0.18 | 0.61 ± 0.36 |
|  | **GBM** | 0.22 ± 0.09 | 0.65 ± 0.38 | 0.48 ± 0.26 | 0.55 ± 0.34 |

Supplementary Table 3. Root Mean Square Error (RMSE) and R^2^ of model predictions of the clinical disability scales per dominant and non-dominant hands among the MS patients at the difficulty level 1. Models are build using 5-fold cross-validation (CV) with 10 repetitions. Results are provided using mean ± SD where the mean and SD are the mean and Standard Deviation across CV repetitions. ElasticNet, SVR Radial, RF, and GBM represent respectively the Elastic net, Support Vector Regression with Radial Basis Function kernel, Random Forest, and Stochastic Gradient Boosting regression models. NA indicate cannot be computed.

| **Outcome variable** | **Models** | **Dominant hand** | | **Non-dominant hand** | |
| --- | --- | --- | --- | --- | --- |
|  |  | **RMSE** | **R^2^** | **RMSE** | **R^2^** |
| **9HPT Average (C1)** | **ElasticNet** | 0.29 ± 0.04 | 0.17 ± 0.07 | 0.29 ± 0.04 | 0.18 ± 0.07 |
|  | **SVR Radial** | 0.29 ± 0.06 | 0.19 ± 0.09 | 0.30 ± 0.05 | 0.19 ± 0.14 |
|  | **RF** | 0.28 ± 0.05 | 0.23 ± 0.08 | 0.29 ± 0.04 | 0.18 ± 0.08 |
|  | **GBM** | 0.29 ± 0.04 | 0.19 ± 0.10 | 0.30 ± 0.05 | 0.13 ± 0.07 |
| **EDSS (C4)** | **ElasticNet** | 0.14 ± 0.01 | 0.08 ± 0.07 | 0.14 ± 0.01 | 0.04 ± 0.05 |
|  | **SVR Radial** | 0.14 ± 0.01 | 0.08 ± 0.06 | 0.14 ± 0.01 | 0.05 ± 0.04 |
|  | **RF** | 0.14 ± 0.02 | 0.04 ± 0.04 | 0.14 ± 0.01 | 0.07 ± 0.07 |
|  | **GBM** | 0.14 ± 0.02 | 0.06 ± 0.04 | 0.14 ± 0.01 | 0.05 ± 0.05 |
| **CombiWISE (C5)** | **ElasticNet** | 0.15 ± 0.01 | 0.12 ± 0.07 | 0.15 ± 0.01 | 0.07 ± 0.05 |
|  | **SVR Radial** | 0.15 ± 0.01 | 0.13 ± 0.08 | 0.15 ± 0.01 | 0.09 ± 0.08 |
|  | **RF** | 0.15 ± 0.01 | 0.07 ± 0.04 | 0.15 ± 0.01 | 0.11 ± 0.06 |
|  | **GBM** | 0.15 ± 0.01 | 0.10 ± 0.06 | 0.15 ± 0.01 | 0.08 ± 0.06 |
| **NeurEx (C6)** | **ElasticNet** | 0.26 ± 0.02 | 0.11 ± 0.06 | 0.27 ± 0.02 | 0.05 ± 0.05 |
|  | **SVR Radial** | 0.27 ± 0.02 | 0.11 ± 0.07 | 0.27 ± 0.02 | 0.06 ± 0.05 |
|  | **RF** | 0.27 ± 0.02 | 0.07 ± 0.05 | 0.27 ± 0.02 | 0.09 ± 0.06 |
|  | **GBM** | 0.27 ± 0.02 | 0.09 ± 0.06 | 0.27 ± 0.02 | 0.09 ± 0.06 |

Supplementary Table 4. The out-of-sample test performance of the clinical disability scales versus the top four most significant spiral derived features (Kurtosis of velocity, radial velocity, angular velocity, and the sum of Hausdorff distances). The test performance was measured using Root Mean Square Error (RMSE) and R^2^ of model predictions per dominant and non-dominant hands among the MS cohorts at the difficulty level 1. ElasticNet, SVR Radial, RF, and GBM represent respectively the Elastic net, Support Vector Regression with Radial Basis Function kernel, Random Forest, and Stochastic Gradient Boosting regression models. NA indicate cannot be computed.

| **Outcome variable** | **Models** | **Dominant hand** | | **Non-dominant hand** | |
| --- | --- | --- | --- | --- | --- |
|  |  | **RMSE** | **R^2^** | **RMSE** | **R^2^** |
| **9HPT Average (C1)** | **ElasticNet** | 0.1507 | 0.1914 | 0.1711 | 0.0474 |
|  | **SVR Radial** | 0.1287 | 0.2243 | 0.1880 | 0.0387 |
|  | **RF** | 0.1811 | 0.1436 | 0.1749 | 0.0744 |
|  | **GBM** | 0.1937 | 0.0752 | 0.1874 | 0.0350 |
| **EDSS (C4)** | **ElasticNet** | 0.1896 | 0.0245 | 0.1923 | 0.0146 |
|  | **SVR Radial** | 0.1962 | 0.0212 | 0.1968 | 0.0229 |
|  | **RF** | 0.1897 | 0.0328 | 0.1953 | 0.0049 |
|  | **GBM** | 0.1923 | 0.0127 | 0.1960 | 0.0027 |
| **CombiWISE (C5)** | **ElasticNet** | 0.2038 | 0.0503 | 0.2082 | 0.0241 |
|  | **SVR Radial** | 0.2058 | 0.0697 | 0.2103 | 0.0492 |
|  | **RF** | 0.2035 | 0.0638 | 0.2090 | 0.0209 |
|  | **GBM** | 0.2063 | 0.0348 | 0.2080 | 0.0323 |
| **NeurEx (C6)** | **ElasticNet** | 0.4195 | 0.0489 | 0.4299 | 0.0195 |
|  | **SVR Radial** | 0.4293 | 0.0757 | 0.4355 | 0.0396 |
|  | **RF** | 0.4177 | 0.0716 | 0.4290 | 0.0236 |
|  | **GBM** | 0.4268 | 0.0292 | 0.4289 | 0.0267 |

Supplementary Table 5. Root Mean Square Error (RMSE) and R^2^ of model predictions of the clinical disability scales per dominant and non-dominant hands among the healthy donors at the difficulty level 2. Models are build using 5-fold cross-validation (CV) with 10 repetitions. Results are provided using mean ± SD where the mean and SD are the mean and Standard Deviation across CV repetitions. ElasticNet, SVR Radial, RF, and GBM represent respectively the Elastic net, Support Vector Regression with Radial Basis Function kernel, Random Forest, and Stochastic Gradient Boosting regression models.

| **Outcome variable** | **Models** | **Dominant hand** | | **Non-dominant hand** | |
| --- | --- | --- | --- | --- | --- |
|  |  | **RMSE** | **R^2^** | **RMSE** | **R^2^** |
| **9HPT Average (C1)** | **ElasticNet** | 0.03 ± 0.01 | 0.55 ± 0.30 | 0.02 ± 0.01 | 0.59 ± 0.32 |
|  | **SVR Radial** | 0.02 ± 0.01 | 0.76 ± 0.28 | 0.02 ± 0.01 | 0.64 ± 0.31 |
|  | **RF** | 0.02 ± 0.01 | 0.63 ± 0.32 | 0.02 ± 0.01 | 0.67 ± 0.31 |
|  | **GBM** | 0.03 ± 0.01 | 0.33 ± 0.31 | 0.03 ± 0.01 | 0.64 ± 0.29 |
| **EDSS (C4)** | **ElasticNet** | 0.22 ± 0.11 | 0.46 ± 0.30 | 0.21 ± 0.05 | 0.56 ± 0.34 |
|  | **SVR Radial** | 0.16 ± 0.08 | 0.59 ± 0.35 | 0.15 ± 0.08 | 0.71 ± 0.29 |
|  | **RF** | 0.15 ± 0.05 | 0.59 ± 0.37 | 0.15 ± 0.05 | 0.62 ± 0.37 |
|  | **GBM** | 0.21 ± 0.12 | 0.40 ± 0.34 | 0.17 ± 0.06 | 0.62 ± 0.36 |
| **CombiWISE (C5)** | **ElasticNet** | 0.22 ± 0.12 | 0.58 ± 0.32 | 0.21 ± 0.12 | 0.61 ± 0.32 |
|  | **SVR Radial** | 0.13 ± 0.04 | 0.56 ± 0.38 | 0.15 ± 0.07 | 0.70 ± 0.31 |
|  | **RF** | 0.14 ± 0.06 | 0.65 ± 0.36 | 0.14 ± 0.05 | 0.62 ± 0.38 |
|  | **GBM** | 0.19 ± 0.09 | 0.47 ± 0.37 | 0.16 ± 0.06 | 0.56 ± 0.37 |
| **NeurEx (C6)** | **ElasticNet** | 0.51 ± 0.18 | 0.52 ± 0.32 | 0.39 ± 0.13 | 0.59 ± 0.37 |
|  | **SVR Radial** | 0.47 ± 0.17 | 0.55 ± 0.33 | 0.29 ± 0.16 | 0.71 ± 0.35 |
|  | **RF** | 0.43 ± 0.08 | 0.50 ± 0.35 | 0.30 ± 0.11 | 0.73 ± 0.30 |
|  | **GBM** | 0.54 ± 0.19 | 0.46 ± 0.35 | 0.36 ± 0.18 | 0.60 ± 0.38 |

Supplementary Table 6. Root Mean Square Error (RMSE) and R^2^ of model predictions of the clinical disability scales per dominant and non-dominant hands among the MS patients at the difficulty level 2. Models are build using 5-fold cross-validation (CV) with 10 repetitions. Results are provided using mean ± SD where the mean and SD are the mean and Standard Deviation across CV repetitions. ElasticNet, SVR Radial, RF, and GBM represent respectively the Elastic net, Support Vector Regression with Radial Basis Function kernel, Random Forest, and Stochastic Gradient Boosting regression models.

| **Outcome variable** | **Models** | **Dominant hand** | | **Non-dominant hand** | |
| --- | --- | --- | --- | --- | --- |
|  |  | **RMSE** | **R^2^** | **RMSE** | **R^2^** |
| **9HPT Average (C1)** | **ElasticNet** | 0.29 ± 0.05 | 0.15 ± 0.06 | 0.30 ± 0.05 | 0.14 ± 0.06 |
|  | **SVR Radial** | 0.30 ± 0.05 | 0.18 ± 0.11 | 0.31 ± 0.06 | 0.13 ± 0.08 |
|  | **RF** | 0.30 ± 0.05 | 0.16 ± 0.10 | 0.30 ± 0.05 | 0.15 ± 0.06 |
|  | **GBM** | 0.30 ± 0.06 | 0.13 ± 0.08 | 0.30 ± 0.04 | 0.15 ± 0.08 |
| **EDSS (C4)** | **ElasticNet** | 0.13 ± 0.01 | 0.09 ± 0.06 | 0.14 ± 0.01 | 0.03 ± 0.03 |
|  | **SVR Radial** | 0.14 ± 0.01 | 0.11 ± 0.07 | 0.14 ± 0.01 | 0.07 ± 0.07 |
|  | **RF** | 0.14 ± 0.01 | 0.06 ± 0.05 | 0.14 ± 0.01 | 0.06 ± 0.05 |
|  | **GBM** | 0.14 ± 0.01 | 0.09 ± 0.06 | 0.14 ± 0.01 | 0.07 ± 0.05 |
| **CombiWISE (C5)** | **ElasticNet** | 0.15 ± 0.01 | 0.13 ± 0.07 | 0.15 ± 0.01 | 0.06 ± 0.05 |
|  | **SVR Radial** | 0.15 ± 0.01 | 0.15 ± 0.09 | 0.15 ± 0.01 | 0.09 ± 0.06 |
|  | **RF** | 0.15 ± 0.01 | 0.09 ± 0.07 | 0.16 ± 0.01 | 0.07 ± 0.06 |
|  | **GBM** | 0.15 ± 0.01 | 0.12 ± 0.06 | 0.15 ± 0.02 | 0.10 ± 0.06 |
| **NeurEx (C6)** | **ElasticNet** | 0.26 ± 0.02 | 0.13 ± 0.07 | 0.27 ± 0.02 | 0.05 ± 0.04 |
|  | **SVR Radial** | 0.26 ± 0.02 | 0.14 ± 0.08 | 0.27 ± 0.02 | 0.08 ± 0.05 |
|  | **RF** | 0.27 ± 0.02 | 0.09 ± 0.06 | 0.27 ± 0.02 | 0.09 ± 0.06 |
|  | **GBM** | 0.26 ± 0.02 | 0.14 ± 0.07 | 0.27 ± 0.02 | 0.10 ± 0.07 |

Supplementary Table 7. The out-of-sample test performance of the clinical disability scales versus the top four most significant spiral derived features (Kurtosis of velocity, radial velocity, angular velocity, and the sum of Hausdorff distances). The test performance was measured using Root Mean Square Error (RMSE) and R^2^ of model predictions per dominant and non-dominant hands among the MS cohorts at the difficulty level 2. ElasticNet, SVR Radial, RF, and GBM represent respectively the Elastic net, Support Vector Regression with Radial Basis Function kernel, Random Forest, and Stochastic Gradient Boosting regression models.

| **Outcome variable** | **Models** | **Dominant hand** | | **Non-dominant hand** | |
| --- | --- | --- | --- | --- | --- |
|  |  | **RMSE** | **R^2^** | **RMSE** | **R^2^** |
| **9HPT Average (C1)** | **ElasticNet** | 0.1554 | 0.1823 | 0.1662 | 0.0621 |
|  | **SVR Radial** | 0.1674 | 0.1724 | 0.1528 | 0.0304 |
|  | **RF** | 0.1844 | 0.1232 | 0.1893 | 0.0518 |
|  | **GBM** | 0.1691 | 0.1556 | 0.1769 | 0.0342 |
| **EDSS (C4)** | **ElasticNet** | 0.1911 | 0.0154 | 0.1929 | 0.0015 |
|  | **SVR Radial** | 0.1972 | 0.0211 | 0.2006 | 0.0119 |
|  | **RF** | 0.1895 | 0.0400 | 0.1916 | 0.0188 |
|  | **GBM** | 0.1891 | 0.0455 | 0.1932 | 0.0073 |
| **CombiWISE (C5)** | **ElasticNet** | 0.2073 | 0.0281 | 0.2076 | 0.0203 |
|  | **SVR Radial** | 0.2085 | 0.0352 | 0.2112 | 0.0422 |
|  | **RF** | 0.2043 | 0.0604 | 0.2049 | 0.0500 |
|  | **GBM** | 0.2078 | 0.0417 | 0.2073 | 0.0281 |
| **NeurEx (C6)** | **ElasticNet** | 0.4272 | 0.0179 | 0.4304 | 0.0035 |
|  | **SVR Radial** | 0.4370 | 0.0254 | 0.4348 | 0.0460 |
|  | **RF** | 0.4225 | 0.0551 | 0.4231 | 0.0410 |
|  | **GBM** | 0.4227 | 0.0569 | 0.4262 | 0.0258 |

Supplementary Table 8. Root Mean Square Error (RMSE) and R^2^ of model predictions of the clinical disability scales per dominant and non-dominant hands among the healthy donors at the difficulty level 3. Models are build using 5-fold cross-validation (CV) with 10 repetitions. Results are provided using mean ± SD where the mean and SD are the mean and Standard Deviation across CV repetitions. ElasticNet, SVR Radial, RF, and GBM represent respectively the Elastic net, Support Vector Regression with Radial Basis Function kernel, Random Forest, and Stochastic Gradient Boosting regression models. NA indicates cannot be computed.

| **Outcome variable** | **Models** | **Dominant hand** | | **Non-dominant hand** | |
| --- | --- | --- | --- | --- | --- |
|  |  | **RMSE** | **R^2^** | **RMSE** | **R^2^** |
| **9HPT Average (C1)** | **ElasticNet** | 0.03 ± 0.01 | 0.45 ± 0.33 | 0.02 ± 0.01 | 0.64 ± 0.31 |
|  | **SVR Radial** | 0.03 ± 0.01 | 0.56 ± 0.34 | 0.03 ± 0.01 | 0.53 ± 0.38 |
|  | **RF** | 0.03 ± 0.01 | 0.55 ± 0.36 | 0.03 ± 0.01 | 0.63 ± 0.28 |
|  | **GBM** | 0.03 ± 0.01 | 0.40 ± 0.29 | 0.03 ± 0.01 | 0.52 ± 0.33 |
| **EDSS (C4)** | **ElasticNet** | 0.23 ± 0.13 | NA | 0.24 ± 0.12 | NA |
|  | **SVR Radial** | 0.22 ± 0.15 | 0.36 ± 0.34 | 0.21 ± 0.15 | 0.41 ± 0.38 |
|  | **RF** | 0.27 ± 0.13 | 0.39 ± 0.33 | 0.27 ± 0.13 | 0.39 ± 0.32 |
|  | **GBM** | 0.30 ± 0.14 | 0.40 ± 0.31 | 0.31 ± 0.14 | 0.49 ± 0.31 |
| **CombiWISE (C5)** | **ElasticNet** | 0.23 ± 0.13 | NA | 0.24 ± 0.12 | NA |
|  | **SVR Radial** | 0.22 ± 0.15 | 0.36 ± 0.34 | 0.21 ± 0.15 | 0.41 ± 0.38 |
|  | **RF** | 0.27 ± 0.13 | 0.39 ± 0.33 | 0.27 ± 0.13 | 0.39 ± 0.32 |
|  | **GBM** | 0.30 ± 0.14 | 0.40 ± 0.31 | 0.31 ± 0.14 | 0.49 ± 0.31 |
| **NeurEx (C6)** | **ElasticNet** | 0.54 ± 0.26 | NA | 0.57 ± 0.24 | NA |
|  | **SVR Radial** | 0.52 ± 0.29 | 0.37 ± 0.32 | 0.50 ± 0.27 | 0.50 ± 0.38 |
|  | **RF** | 0.60 ± 0.21 | 0.30 ± 0.32 | 0.62 ± 0.23 | 0.37 ± 0.34 |
|  | **GBM** | 0.67 ± 0.33 | 0.42 ± 0.34 | 0.70 ± 0.30 | 0.41 ± 0.33 |

Supplementary Table 9. Root Mean Square Error (RMSE) and R^2^ of model predictions of the clinical disability scales per dominant and non-dominant hands among the multiple sclerosis patients at the difficulty level 3. Models are build using 5-fold cross-validation (CV) with 10 repetitions. Results are provided using mean ± SD where the mean and SD are the mean and Standard Deviation across CV repetitions. ElasticNet, SVR Radial, RF, and GBM represent respectively the Elastic net, Support Vector Regression with Radial Basis Function kernel, Random Forest, and Stochastic Gradient Boosting regression models. NA indicates cannot be computed.

| **Outcome variable** | **Models** | **Dominant hand** | | **Non-dominant hand** | |
| --- | --- | --- | --- | --- | --- |
|  |  | **RMSE** | **R^2^** | **RMSE** | **R^2^** |
| **9HPT Average (C1)** | **ElasticNet** | 0.31 ± 0.05 | 0.12 ± 0.06 | 0.33 ± 0.04 | 0.12 ± 0.09 |
|  | **SVR Radial** | 0.32 ± 0.05 | 0.14 ± 0.09 | 0.32 ± 0.05 | 0.19 ± 0.13 |
|  | **RF** | 0.31 ± 0.04 | 0.17 ± 0.09 | 0.33 ± 0.05 | 0.11 ± 0.09 |
|  | **GBM** | 0.32 ± 0.04 | 0.11 ± 0.06 | 0.34 ± 0.05 | 0.07 ± 0.06 |
| **EDSS (C4)** | **ElasticNet** | 0.14 ± 0.01 | 0.04 ± 0.04 | 0.14 ± 0.01 | 0.03 ± 0.03 |
|  | **SVR Radial** | 0.14 ± 0.01 | 0.11 ± 0.08 | 0.14 ± 0.01 | 0.09 ± 0.06 |
|  | **RF** | 0.14 ± 0.01 | 0.07 ± 0.06 | 0.14 ± 0.01 | 0.04 ± 0.05 |
|  | **GBM** | 0.13 ± 0.01 | 0.10 ± 0.07 | 0.14 ± 0.01 | 0.06 ± 0.05 |
| **CombiWISE (C5)** | **ElasticNet** | 0.16 ± 0.01 | 0.05 ± 0.05 | 0.16 ± 0.01 | 0.03 ± 0.03 |
|  | **SVR Radial** | 0.15 ± 0.01 | 0.12 ± 0.08 | 0.16 ± 0.01 | 0.10 ± 0.06 |
|  | **RF** | 0.15 ± 0.01 | 0.11 ± 0.08 | 0.16 ± 0.01 | 0.05 ± 0.04 |
|  | **GBM** | 0.15 ± 0.01 | 0.10 ± 0.07 | 0.16 ± 0.01 | 0.06 ± 0.06 |
| **NeurEx (C6)** | **ElasticNet** | 0.27 ± 0.02 | 0.04 ± 0.04 | 0.28 ± 0.02 | 0.03 ± 0.03 |
|  | **SVR Radial** | 0.26 ± 0.02 | 0.13 ± 0.07 | 0.28 ± 0.02 | 0.09 ± 0.07 |
|  | **RF** | 0.27 ± 0.02 | 0.10 ± 0.05 | 0.28 ± 0.02 | 0.05 ± 0.04 |
|  | **GBM** | 0.27 ± 0.02 | 0.10 ± 0.05 | 0.28 ± 0.02 | 0.06 ± 0.04 |

Supplementary Table 10. The out-of-sample test performance of the clinical disability scales versus the top four most significant spiral derived features (Kurtosis of velocity, radial velocity, angular velocity, and the sum of Hausdorff distances). The test performance was measured using Root Mean Square Error (RMSE) and R^2^ of model predictions per dominant and non-dominant hands among the multiple sclerosis cohorts at the difficulty level 3. ElasticNet, SVR Radial, RF, and GBM represent respectively the Elastic net, Support Vector Regression with Radial Basis Function kernel, Random Forest, and Stochastic Gradient Boosting regression models. NA indicates cannot be computed.

| **Outcome variable** | **Models** | **Dominant hand** | | **Non-dominant hand** | |
| --- | --- | --- | --- | --- | --- |
|  |  | **RMSE** | **R^2^** | **RMSE** | **R^2^** |
| **9HPT Average (C1)** | **ElasticNet** | 0.1645 | 0.0868 | 0.1894 | 0.0347 |
|  | **SVR Radial** | 0.1522 | 0.1243 | 0.1487 | 0.0734 |
|  | **RF** | 0.2155 | 0.0740 | 0.1836 | 0.0539 |
|  | **GBM** | 0.1964 | 0.0933 | 0.1709 | 0.0688 |
| **EDSS (C4)** | **ElasticNet** | 0.1921 | 0.0068 | 0.1934 | 0.0030 |
|  | **SVR Radial** | 0.1973 | 0.0367 | 0.1990 | 0.0171 |
|  | **RF** | 0.1863 | 0.0751 | 0.1921 | 0.0207 |
|  | **GBM** | 0.1888 | 0.0513 | 0.1921 | 0.0219 |
| **CombiWISE (C5)** | **ElasticNet** | 0.2100 | 0.0121 | 0.2103 | 0.0105 |
|  | **SVR Radial** | 0.2117 | 0.0451 | 0.2129 | 0.0251 |
|  | **RF** | 0.2025 | 0.0967 | 0.2098 | 0.0239 |
|  | **GBM** | 0.2073 | 0.0558 | 0.2105 | 0.0163 |
| **NeurEx (C6)** | **ElasticNet** | 0.4322 | 0.0099 | 0.4350 | 0.0054 |
|  | **SVR Radial** | 0.4369 | 0.0463 | 0.4423 | 0.0254 |
|  | **RF** | 0.4188 | 0.0969 | 0.4314 | 0.0216 |
|  | **GBM** | 0.4271 | 0.0489 | 0.4319 | 0.0219 |

Supplementary Table 11. The out-of-sample test performance of the clinical disability scales versus the sum of Hausdorff distances (HDis) using the linear regression model. The test performance was measured using Root Mean Square Error (RMSE) and R^2^ of model predictions per dominant and non-dominant hands among the multiple sclerosis cohort at the difficulty level 1, 2, and 3.

| **Model variables** | **Difficulty Levels** | **Dominant hand** | | **Non-dominant hand** | |
| --- | --- | --- | --- | --- | --- |
|  |  | **RMSE** | **R^2^** | **RMSE** | **R^2^** |
| **9HPT Average (C1)**  **Vs.**  **Sum of HDis (F24)** | **1** | 0.1599 | 0.1589 | 0.1750 | 0.0817 |
|  | **2** | 0.1611 | 0.1593 | 0.1692 | 0.0654 |
|  | **3** | 0.1622 | 0.1154 | 0.1741 | 0.0774 |
| **EDSS (C4)**  **Vs.**  **Sum of HDis (F24)** | **1** | 0.1889 | 0.0302 | 0.1904 | 0.0125 |
|  | **2** | 0.1904 | 0.0166 | 0.1909 | 0.0083 |
|  | **3** | 0.1917 | 0.0105 | 0.1927 | 0.0099 |
| **CombiWISE (C5)**  **Vs.**  **Sum of HDis (F24)** | **1** | 0.2038 | 0.0502 | 0.2061 | 0.0246 |
|  | **2** | 0.2063 | 0.0311 | 0.2068 | 0.0186 |
|  | **3** | 0.2085 | 0.0177 | 0.2102 | 0.0220 |
| **NeurEx (C6)**  **Vs.**  **Sum of HDis (F24)** | **1** | 0.4217 | 0.0404 | 0.4250 | 0.0187 |
|  | **2** | 0.4258 | 0.0229 | 0.4265 | 0.0126 |
|  | **3** | 0.4308 | 0.0112 | 0.4348 | 0.0110 |

Supplementary Table 12. Root Mean Square Error (RMSE) and R^2^ of model predictions of the clinical disability scales per dominant and non-dominant hands among the multiple sclerosis patients at the difficulty level 1 while controlling for the age and gender variables in the model. Models are build using 5-fold cross-validation (CV) with 10 repetitions. Results are provided using mean ± SD where the mean and SD are the mean and Standard Deviation across CV repetitions. ElasticNet, SVR Radial, RF, and GBM represent respectively the Elastic net, Support Vector Regression with Radial Basis Function kernel, Random Forest, and Stochastic Gradient Boosting regression models. NA indicate cannot be computed.

| **Outcome variable** | **Models** | **Dominant hand** | | **Non-dominant hand** | |
| --- | --- | --- | --- | --- | --- |
|  |  | **RMSE** | **R^2^** | **RMSE** | **R^2^** |
| **9HPT Average (C1)** | **ElasticNet** | 0.29 ± 0.05 | 0.18 ± 0.07 | 0.29 ± 0.04 | 0.20 ± 0.08 |
|  | **SVR Radial** | 0.29 ± 0.06 | 0.20 ± 0.09 | 0.26 ± 0.05 | 0.35 ± 0.17 |
|  | **RF** | 0.27 ± 0.06 | 0.26 ± 0.09 | 0.27 ± 0.05 | 0.30 ± 0.13 |
|  | **GBM** | 0.28 ± 0.04 | 0.26 ± 0.12 | 0.28 ± 0.06 | 0.23 ± 0.12 |
| **EDSS (C4)** | **ElasticNet** | 0.13 ± 0.01 | 0.15 ± 0.06 | 0.14 ± 0.01 | 0.08 ± 0.04 |
|  | **SVR Radial** | 0.13 ± 0.01 | 0.18 ± 0.08 | 0.12 ± 0.01 | 0.26 ± 0.11 |
|  | **RF** | 0.11 ± 0.01 | 0.36 ± 0.12 | 0.12 ± 0.01 | 0.32 ± 0.14 |
|  | **GBM** | 0.12 ± 0.01 | 0.27 ± 0.12 | 0.12 ± 0.01 | 0.25 ± 0.11 |
| **CombiWISE (C5)** | **ElasticNet** | 0.15 ± 0.01 | 0.16 ± 0.06 | 0.15 ± 0.01 | 0.10 ± 0.06 |
|  | **SVR Radial** | 0.14 ± 0.01 | 0.19 ± 0.10 | 0.14 ± 0.01 | 0.26 ± 0.12 |
|  | **RF** | 0.13 ± 0.02 | 0.29 ± 0.11 | 0.13 ± 0.01 | 0.31 ± 0.12 |
|  | **GBM** | 0.14 ± 0.01 | 0.23 ± 0.09 | 0.14 ± 0.01 | 0.21 ± 0.10 |
| **NeurEx (C6)** | **ElasticNet** | 0.25 ± 0.02 | 0.19 ± 0.09 | 0.26 ± 0.02 | 0.11 ± 0.07 |
|  | **SVR Radial** | 0.25 ± 0.01 | 0.22 ± 0.07 | 0.24 ± 0.02 | 0.29 ± 0.11 |
|  | **RF** | 0.23 ± 0.03 | 0.31 ± 0.11 | 0.23 ± 0.02 | 0.32 ± 0.11 |
|  | **GBM** | 0.24 ± 0.03 | 0.27 ± 0.09 | 0.25 ± 0.02 | 0.26 ± 0.11 |

Supplementary Table 13. The out-of-sample test performance of the clinical disability scales versus the top four most significant spiral derived features (Kurtosis of velocity, radial velocity, angular velocity, and the sum of Hausdorff distances) while controlling for the age and gender variables in the model. The test performance was measured using Root Mean Square Error (RMSE) and R^2^ of model predictions per dominant and non-dominant hands among the multiple sclerosis cohort at the difficulty level 1. ElasticNet, SVR Radial, RF, and GBM represent respectively the Elastic net, Support Vector Regression with Radial Basis Function kernel, Random Forest, and Stochastic Gradient Boosting regression models. NA indicate cannot be computed.

| **Outcome variable** | **Models** | **Dominant hand** | | **Non-dominant hand** | |
| --- | --- | --- | --- | --- | --- |
|  |  | **RMSE** | **R^2^** | **RMSE** | **R^2^** |
| **9HPT Average (C1)** | **ElasticNet** | 0.1436 | 0.2746 | 0.1554 | 0.1448 |
|  | **SVR Radial** | 0.1214 | 0.2689 | 0.1495 | 0.1189 |
|  | **RF** | 0.1585 | 0.2240 | 0.1565 | 0.1052 |
|  | **GBM** | 0.2435 | 0.1249 | 0.1568 | 0.1255 |
| **EDSS (C4)** | **ElasticNet** | 0.1794 | 0.1359 | 0.1836 | 0.1105 |
|  | **SVR Radial** | 0.1802 | 0.1441 | 0.1982 | 0.0439 |
|  | **RF** | 0.1921 | 0.0528 | 0.2061 | 0.0041 |
|  | **GBM** | 0.1896 | 0.0695 | 0.2071 | 0.0048 |
| **CombiWISE (C5)** | **ElasticNet** | 0.1924 | 0.1766 | 0.1993 | 0.1176 |
|  | **SVR Radial** | 0.1936 | 0.1571 | 0.2157 | 0.0440 |
|  | **RF** | 0.1999 | 0.1019 | 0.2174 | 0.0157 |
|  | **GBM** | 0.2060 | 0.0711 | 0.2129 | 0.0315 |
| **NeurEx (C6)** | **ElasticNet** | 0.3962 | 0.1914 | 0.4109 | 0.1347 |
|  | **SVR Radial** | 0.3881 | 0.2157 | 0.4121 | 0.0738 |
|  | **RF** | 0.3946 | 0.1812 | 0.4284 | 0.0445 |
|  | **GBM** | 0.4036 | 0.1585 | 0.4340 | 0.0342 |

# R code

All R code and data used to complete the analysis have been made available at <https://github.com/bielekovaLab/Bielekova-Lab-Code/tree/master/FormerLabMembers/Messan_Komi>.

**References**

Aljanabi, M. A., Hussain, Z. M., & Lu, S. F. (2018). An Entropy-Histogram Approach for Image Similarity and Face Recognition. *Mathematical Problems in Engineering, 2018*. doi:Artn 9801308

10.1155/2018/9801308

Asamoah, D., Ofori, E., Opoku, S., & Danso, J. (2018). Measuring the Performance of Image Contrast Enhancement Technique. *International Journal of Computer Applications, 181*(22), 6-13. doi:10.5120/ijca2018917899

Creagh, A. P., Simillion, C., Scotland, A., Lipsmeier, F., Bernasconi, C., Belachew, S., . . . De Vos, M. (2020). Smartphone-based remote assessment of upper extremity function for multiple sclerosis using the Draw a Shape Test. *Physiological Measurement, 41*(5), 054002. doi:10.1088/1361-6579/ab8771

Dubuisson, M.-P., & Jain, A. K. (1994). A modified Hausdorff distance for object matching. *Proceedings of 12th international conference on pattern recognition, IEEE 1*, 566-568.

Erasmus, L. P., Sarno, S., Albrecht, H., Schwecht, M., Pollmann, W., & Konig, N. (2001). Measurement of ataxic symptoms with a graphic tablet: standard values in controls and validity in Multiple Sclerosis patients. *J Neurosci Methods, 108*(1), 25-37. doi:10.1016/s0165-0270(01)00373-9

Huttenlocher, D. P., Klanderman, G. A., & Rucklidge, W. J. (1993). Comparing images using the hausdorff distance. *IEEE Transactions on pattern analysis and machine intelligence, 15*, 850-863.

Memedi, M., Aghanavesi, S., & Westin, J. (2016). A method for measuring Parkinson’s disease related temporal irregularity in spiral drawings. *2016 IEEE-EMBS International Conference on Biomedical and Health Informatics (BHI)*, 410-413.

Memedi, M., Sadikov, A., Groznik, V., Zabkar, J., Mozina, M., Bergquist, F., . . . Nyholm, D. (2015). Automatic Spiral Analysis for Objective Assessment of Motor Symptoms in Parkinson's Disease. *Sensors (Basel), 15*(9), 23727-23744. doi:10.3390/s150923727

Tsai, D. Y., Lee, Y., & Matsuyama, E. (2008). Information entropy measure for evaluation of image quality. *J Digit Imaging, 21*(3), 338-347. doi:10.1007/s10278-007-9044-5
